# Aptamer-based optical manipulation of protein subcellular localization in cells

**DOI:** 10.1038/s41467-020-15113-2

**Published:** 2020-03-12

**Authors:** Sitao Xie, Yulin Du, Yu Zhang, Zhimin Wang, Dailiang Zhang, Lei He, Liping Qiu, Jianhui Jiang, Weihong Tan

**Affiliations:** 1 Molecular Science and Biomedicine Laboratory (MBL), State Key Laboratory of Chemo/Bio-Sensing and Chemometrics, College of Chemistry and Chemical Engineering, College of Biology, Collaborative Innovation Center for Chemistry and Molecular Medicine, Hunan University, Changsha, 410082 China; 2 Institute of Cancer and Basic Medicine (IBMC), Chinese Academy of Sciences, The Cancer Hospital of the University of Chinese Academy of Sciences, Hangzhou, Zhejiang 310022 China; 3 Institute of Molecular Medicine (IMM), Renji Hospital, State Key Laboratory of Oncogenes and Related Genes, Shanghai Jiao Tong University School of Medicine, and College of Chemistry and Chemical Engineering, Shanghai Jiao Tong University, Shanghai, 200240 China

**Keywords:** Chemical tools, Intracellular signalling peptides and proteins, Chemical tools, Proteins, Intracellular signalling peptides and proteins

## Abstract

Protein-dominant cellular processes cannot be fully decoded without precise manipulation of their activity and localization in living cells. Advances in optogenetics have allowed spatiotemporal control over cellular proteins with molecular specificity; however, these methods require recombinant expression of fusion proteins, possibly leading to conflicting results. Instead of modifying proteins of interest, in this work, we focus on design of a tunable recognition unit and develop an aptamer-based near-infrared (NIR) light-responsive nanoplatform for manipulating the subcellular localization of specific proteins in their native states. Our results demonstrate that this nanoplatform allows photocontrol over the cytoplasmic-nuclear shuttling behavior of the target RelA protein (a member of the *NF-κβ* family), enabling regulation of RelA-related signaling pathways. With a modular design, this aptamer-based nanoplatform can be readily extended for the manipulation of different proteins (e.g., lysozyme and p53), holding great potential to develop a variety of label-free protein photoregulation strategies for studying complex biological events.

## Abstract (web summary)

Optogenetic manipulation of protein localisation in cells involves the creation of fusions that can influence activity. Here the authors develop a near-infrared light-responsive aptamer-based system to regulate the nuclear-cytoplasmic shuttling of NF-κB subunit RelA.

## Introduction

Eukaryotic cells are elaborately subdivided into functionally distinct membrane-bound compartments. Dynamically coordinating the activity and subcellular localization of proteins is fundamentally required for various cellular processes, such as signaling, metabolism, polarization, and apoptosis^1,2^. For instance, p53, a principal tumor-suppressor protein, plays an important role in the inhibition of autophagy and induction of apoptosis when localizing in cytoplasm, but participating in DNA replication and cell cycle regulation when being recruited to nucleus^3^. Disorder of subcellular protein translocation underlies many human diseases, such as metabolic diseases, neurodegenerative diseases, and even cancers^4^. Indeed, techniques enabling control over the subcellular translocation of specific proteins with biologically comparable spatial and temporal precision would be critical for elucidating not only the biofunction of individual proteins but also the mechanism of complex biological pathways.

Taking advantage of high spatial and temporal precision, light has been widely used as an external trigger for biological regulation^5,6^. While methods using light-responsive small molecules have been reported for manipulating protein translocation activity^6,7^, their applications were limited by severe off-target effects. By combining techniques from optics and genetics, optogenetics showed feasible for providing both high spatiotemporal resolution and high molecular specificity for controlling protein translocation activity^8–11^. On the other hand, proteins were endowed with photoresponsive directionality through the site-specific genetic fusion of photoreceptors or molecular tags, which could alter natural protein structure. Also, such a time-consuming and complicated genetic manipulation process could perturb the normal cellular pathways^12^. These confounding factors could potentially change the function and behavior of target protein and potentially affect the related biological processes. Therefore, alternative precise protein manipulation strategies with minimal interference on their natural structure/expression would be highly beneficial for studying protein-related cellular processes.

To exert spatiotemporal control over native protein of interest, optically tunable recognition units are potentially useful. Particularly suited to this end, aptamers are single-stranded oligonucleotides screened from a large random sequence pool via in vitro methods^13^ based on their specific recognition for target molecules. By combining their excellent molecular recognition capability with the intrinsic advantage of nucleic acids, including convenient modification^14^, flexible designability^15^, and high programmability^16^, aptamers have attracted widespread attention for extensive biological applications^17–20^, especially for protein regulation^21^. However, most previous works were restricted to test tubes^22^ or the cell surface^23^, while only a few have focused on cellular context beyond the plasma membrane.

**Fig. 1:**
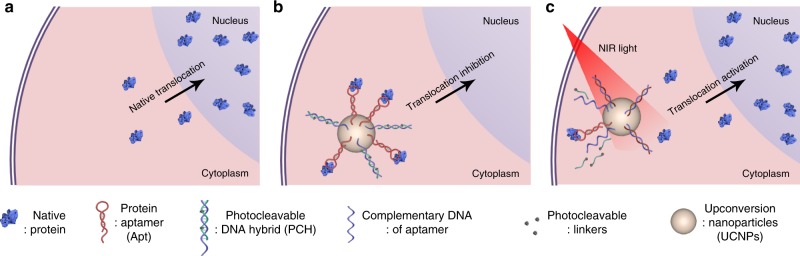
Schematic illustration of the aptamer-based NIR-responsive nanoplatform for manipulating the subcellular localization of native protein in living cells. **a** Translocation behavior of proteins from cytoplasm to nucleus. **b** Protein-specific aptamers (Apt) and photocleavable DNA hybrid (PCH_2_) are functionalized onto the surface of UCNPs. After internalization into the cytoplasm, PHC_2_-Apt-UCNPs can specifically capture target proteins and inhibit their subcellular trafficking behavior. **c** Based on the capability of UCNPs for transforming NIR light excitation into UV light emission, PHC_2_ can be activated to compete Apt from the Apt/protein binding complex upon NIR laser irradiation. In this way, proteins can be released to restore their natural biofunction.

In this work, aiming to achieve precise control over the subcellular localization of specific proteins without altering their natural structure and expression in living cells, we develop an aptamer-based photoresponsive nanoplatform (Fig. 1). Specifically, we chose RelA, an essential transcription factor involved in many cellular processes, as the model protein. RelA is a subunit protein of the nuclear factor-kappa B (*NF-κB*) family, whose cellular distribution is tightly modulated through sophisticated cellular machinery^24,25^. An aptamer (Apt) that could specifically recognize RelA protein with high binding affinity (Kd = 0.64 nM)^26^ is used as the recognition unit. To impart spatiotemporal regulation on target protein, a photoresponsive double-stranded DNA hybrid is designed as the regulation unit for the functional conformation of Apt. One DNA strand is partly complementary to the sequence of Apt (termed as cDNA), while the other strand containing ultraviolet (UV) photocleavable linkers (PC-linkers) serves as a blocking probe (termed as bDNA) for the cDNA. To avoid the biological damage of UV light commonly used in photoregulation systems, upconversion nanoparticles (UCNPs) with the ability to convert NIR excitation into UV light emission is chosen as the “core” of protein aggregation. Apt and the photocleavable cDNA/bDNA hybrid (PCHx, where x represented the number of PC linkers inserted in the sequence of bDNA) are functionalized onto the surface of UCNPs, and the resultant nanoparticle is termed as PCHx-Apt-UCNPs. Owing to higher hybridization stability with intact bDNA, cDNA induces little influence on the molecular recognition capability of Apt. After cellular internalization of PCHx-Apt-UCNPs, target RelA protein can be specifically captured by Apt and assembled around the UCNP core. Our previous work^27^ has demonstrated that formation of high-order protein clusters could efficiently trap proteins and then inhibit their activity. In this way, the subcellular trafficking behavior of RelA is effectively inhibited. Upon excitation with NIR laser, UCNPs can emit UV light to cleave the PC linker, liberating cDNA to compete Apt from the RelA/Apt binding complex. Subsequently, the captured RelA protein can be released to restore its natural activity. By modulating the subcellular translocation behavior of target proteins, the related intracellular signaling pathways could be efficiently modulated, thus providing a potent nanoplatform for studying the mechanism underlying these biological processes.

## Results

### Construction of aptamer-based photoresponsive nanoplatform

To develop a high-performance nanoplatform for protein manipulation, a key consideration was centered on the relative hybridization stability of the cDNA/Apt complex and the cDNA/bDNA complex. Ideally, the cDNA/bDNA hybrid should be stable enough to minimize the interference of cDNA on the binding efficiency of aptamer with target protein. Meanwhile, upon photoactivation, cDNA should be rapidly liberated to compete Apt from the RelA/Apt complex to efficiently release the captured RelA protein. The sequences (Supplementary Table 1) were designed according to the DNA secondary structure and thermodynamic stability (Gibbs free energy, *ΔG*) calculated with the NUPACK software (Supplementary Fig. 1). To ensure minimal leakage, *ΔG* of the cDNA/Apt hybrid (−23.21 kcal mol^−1^) was ~5 kcal mol^−1^ higher than that of the cDNA/bDNA hybrid (−28.22 kcal mol^−1^). Meanwhile, to guarantee rapid photoactivation of protein, *ΔG* of the hybridization between cDNA and the cleaved bDNA fragments should be apparently higher than that of the cDNA/aptamer hybrid (Supplementary Fig. 1b–f). Three bDNAs inserted with different numbers of PC linker (zero, one and two) were designed to optimize the photoregulation efficiency, and the corresponding cDNA/bDNA hybrids were termed as PCH_0_, PCH_1_ and PCH_2_, respectively.

**Fig. 2:**
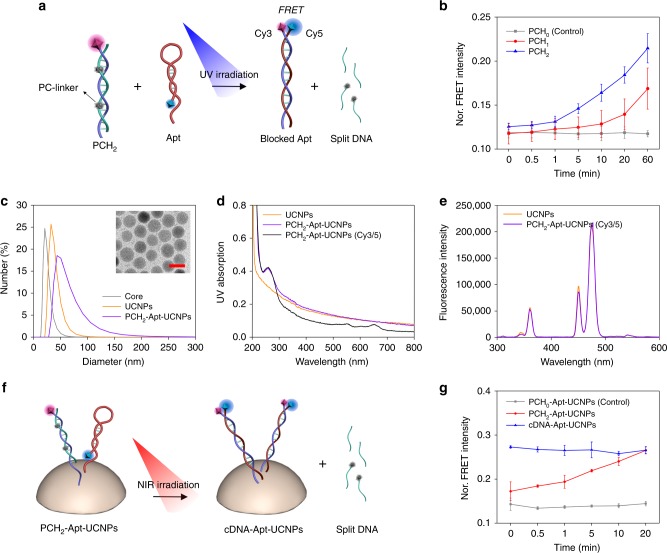
Design and characterization of the aptamer-based light-responsive nanoplatform. **a** Schematic illustration of the strand replacement reaction between Cy5-labeled Apt and Cy3-labeled PCH_2_ under UV light irradiation. **b** Kinetic analysis of the Förster resonance energy transfer (FRET) signal between Cy3 and Cy5 in the case of PCH_0_, PCH_1_, and PCH_2_. Error bars represent the standard deviation of three independent experiments. Data are presented as mean values ± S.D. **c** Dynamic light scattering spectra of the UCNP core, UCNPs and PCH_2_-Apt-UCNPs. Inset: TEM image of the UCNP core. The red scale bar represents 20 nm. **d** UV absorption of UCNPs, PCH_2_-Apt-UCNPs and PCH_2_-Apt-UCNPs (Cy3/5) (PCH_2_-Apt-UCNPs containing the Cy5-labeled Apt and Cy3-labeled PCH_2_). **e** Luminescence spectra of UCNPs and PCH_2_-Apt-UCNPs (Cy3/5) under 980 nm excitation. **f** Schematic illustration of the NIR-responsive hybridization switch of PCH_2_-Apt-UCNPs. **g** Kinetic analysis of the FRET signal of PCH_2_-Apt-UCNPs. Error bars represent the standard deviation of three independent experiments. Data are presented as mean values ± S.D.

To establish a fluorescence signal for assessing the feasibility of our sequence design, a Cy3 fluorophore was modified at the 3′- end of the cDNA, and a Cy5 fluorophore was modified at the 5′-end of Apt. The Förster resonance energy transfer (FRET) signal between Cy3 and Cy5 was used to study the DNA hybridization dynamics. When mixing Apt, cDNA and bDNA together at a ratio of 1:1:1, cDNA preferentially hybridized with bDNA, resulting in a low FRET signal. However, upon irradiation with UV light, the PC linkers were quickly cleaved, liberating cDNA to hybridize with Apt. As such, the Cy3 fluorophore and the Cy5 fluorophore were brought together, leading to the FRET signal enhancement (Fig. 2a). Meanwhile, the FRET signal enhancement was proportional to the time of UV irradiation (Fig. 2b). As expected, for the sample of PCH_1_, the photoresponsive kinetics was apparently lower than that of PCH_2_, while little FRET signal change was observed for PCH_0_, indicating a PC linker-dependent photoresponsive strand displacement reaction. To strike a balance between photoregulation efficiency and the cost of synthesis, PCH_2_ was chosen for the rest of study.

After evaluating the sequence design of these DNA probes, we next used them to fabricate a functionalized nanomanipulator. The synthesized UCNPs displayed a spherical shape with diameter of 22.38 ± 1.27 nm in the transmission electron microscopy (TEM) images (Supplementary Fig. 2). Successful modification of UCNPs with DNA probes was characterized by an enhanced hydrodynamic diameter (Fig. 2c), a reduced Zeta potential from 25.3 ± 0.9 mV to −16.2 ± 0.9 mV (Supplementary Fig. 3), and an apparent peak of nucleic acids (260 nm) in the absorption spectra (Fig. 2d). Meanwhile, the fluorescence spectrum of the resultant DNA-modified UCNPs, PCH_2_-Apt-UCNPs, was similar to that of unmodified UCNPs (Fig. 2e), indicating that DNA modification did not interfere with the optical property of UCNPs. To verify the light-driven hybridization switch of these DNA probes on the surface of UCNPs, UV light irradiation was first applied, and the FRET signal between Cy3 and Cy5 was recorded. As shown in Fig. 2g, the FRET signal of PCH_2_-Apt-UCNPs increased with extension of UV irradiation time and reached saturation after 20 min. On the other hand, only negligible FRET signal change was observed in the control PCH_0_-Apt-UCNPs with no PC linkers. Meanwhile, based on the capability of UCNPs for transforming NIR light excitation to UV light emission, a similar trend toward the change of FRET signal was observed with NIR light irradiation (Supplementary Fig. 4). The FRET signal of PCH_2_-rDNA-UCNPs, wherein the aptamer was replaced with random DNA sequence, exhibited undetectable difference irrespective of light irradiation, revealing that the FRET signal enhancement was originated from Apt/cDNA hybridization (Supplementary Fig. 5). In addition, both nanoparticles and NIR laser irradiation caused negligible impact on cell viability (Supplementary Fig. 6). Taken together, the NIR light-triggered conformation switching of Apt in PCH_2_-Apt-UCNPs was expected to enable specific regulation of RelA protein behavior in living cells.

### Optical manipulation of native protein localization in cells

**Fig. 3:**
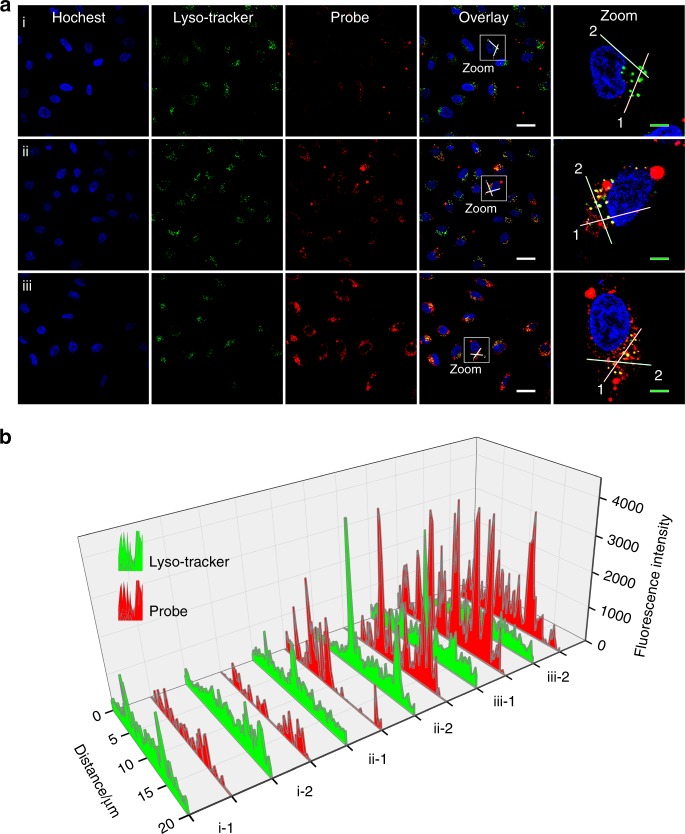
Time-dependent cellular uptake and intracellular distribution of PCH_2_-Apt-UCNPs. **a** Confocal laser scanning microscopy (CLSM) imaging of living A549 cells treated with PCH_2_-Apt-UCNPs for 2 h (i), 4 h (ii) and 6 h (iii). **b** Cy5 fluorescence intensity analysis of the linear regions (i-1,2 ii-1,2 and iii-1,2) in zoom images. The PCH_2_-Apt-UCNPs was traced with the Cy5 fluorescence signal. Nuclei and lysosomes were stained with Hoechst 33258 (blue) and LysoTracker Green (green), respectively. Scale bars in overlay images and zoom images represent 30 μm and 6 μm, respectively.

In a normal state, RelA remains in the cytoplasm, and its activity is inhibited through binding with inhibitory IκB members. However, once activated by some external stimuli, for example, tumor necrosis factor α (TNFα), RelA could rapidly enter the nucleus to trigger a cascade of signaling events^28^. Therefore, to impart precise control over the behavior of RelA protein, it was necessary to deliver a sufficient amount of PCH_2_-Apt-UCNPs in the cytoplasm. A lung carcinoma cell line, A549, was used as the cell model. A549 cells were incubated with PCH_2_-Apt-UCNPs (100 μg mL^−1^) for different length of time. Their cellular uptake and intracellular distribution were monitored from the Cy5 signal of Apt with confocal laser scanning microscopy (CLSM). Accumulation of PCH_2_-Apt-UCNPs in the early endosome was first excluded by their low co-localization with the early endosomal protein marker, EEA, (Supplementary Figs. 7 and 8). Meanwhile, as shown in Fig. 3, the cellular uptake of PCH_2_-Apt-UCNPs was gradually enhanced by increasing the incubation time from 2 to 6 h. As verified by the fluorescence signal of LysoTracker, an indicator of lysosome and late endosome, the lysosomal (or late endosomal) distribution of PCH_2_-Apt-UCNPs was gradually reduced. The fluorescence profiles of the linear region further confirmed that a large proportion of PCH_2_-Apt-UCNPs was translocated from lysosome into cytoplasm after incubation for 6 h (Fig. 3b).

After demonstrating that PCH_2_-Apt-UCNPs could be internalized into the cytoplasm, we tested their potential for regulating the translocation behavior of target protein. To accomplish this, A549 cells were first incubated with PCH_2_-Apt-UCNPs for 6 h and then stimulated with TNFα (2.5 ng mL^−1^) for 1.5 h. The intracellular distribution of RelA was analyzed with fluorescence immunostaining. For the sample treated with PCH_2_-Apt-UCNPs, RelA was mainly stuck in the cytoplasm, even under TNF*α* stimulation; while a significant nuclear recruitment of RelA was observed in the control samples (Supplementary Fig. 9). Neither equivalent UCNPs nor PCH_2_-rDNA-UCNPs induced any observable alteration of the regular trafficking behavior of RelA. Of note, without TNF*α* stimulation, RelA remained in the cytoplasm of the cells treated with UCNPs and/or NIR light (Supplementary Fig. 10), indicating that both UCNPs and NIR irradiation induced little impact on the RelA translocation. These results demonstrated that alteration of RelA cellular distribution originated from aptamer-specific RelA assembly around the UCNP core, which was further confirmed by fluorescence intensity calculation using Image J software (Supplementary Figs. 11 and 12).

**Fig. 4:**
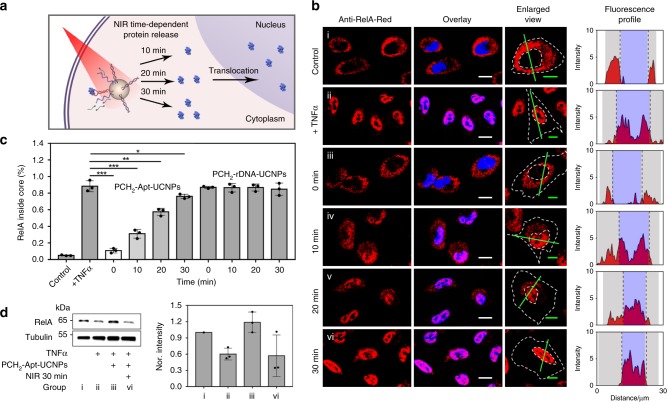
Manipulation of native RelA localization in living A549 cells with NIR light irradiation. **a** Schematic illustration of NIR-dependent nuclear transport of RelA. **b** CLSM imaging of A549 cells with different treatments: untreated control (i), TNF*α* stimulation (ii), PCH_2_-Apt-UCNPs pretreatment, followed by NIR laser irradiation for 0 min (iii), 10 min (iv), 20 min (v) and 30 min (vi) min, and TNFα stimulation. From left to right: fluorescence channel of PE-anti RelA, enlarged view of the PE fluorescence channel, and PE fluorescence profiling of the straight green line (The purple domain represents the nuclear region; the gray domain represents the cytoplasmic region). Scale bars in overlay images and enlarged view images represent 15 μm and 10 μm, respectively. **c** Relative fluorescence intensity of PE-anti RelA in the nuclei of A549 cells with different treatments. For the statistical analysis, data were obtained from three CLSM images and analyzed by using the Image J software for each condition. Error bars represent the standard deviation of three independent experiments. Data are presented as mean values ± S.D. *P = 0.041 ≤ 0.05, **P = 0.0025 ≤ 0.01, ***P = 0.000049 (0.00030) for 0 (10) min group ≤0.001, by two-tailed unpaired Student’s *t*-test. **d** Western blot analysis of native RelA in the cytoplasm of A549 cells after different treatments: untreated control (i), TNF*α* stimulation (ii), and PCH_2_-Apt-UCNPs pretreatment followed by TNF*α* stimulation after NIR laser irradiation for 0 min (iii) or 30 min (vi). Error bars represent the standard deviation of three independent experiments. Data are presented as mean values ± S.D.

We next tested their capacity of PCH_2_-Apt-UCNPs to regulate the subcellular localization of RelA with NIR laser irradiation. A significant nuclear accumulation of RelA was observed, while the RelA signal in cytoplasm was apparently reduced (Fig. 4b). In addition, the nuclear transport of RelA showed an irradiation time-dependent pattern, which was further verified by the fluorescence profile of the designated region (green line in Fig. 4b). Based on fluorescence intensity calculation using CLSM data, the percentage of RelA in nuclei was enhanced by over 70% after a 30-min NIR laser irradiation (Fig. 4c), indicating that the subcellular trafficking behavior of native RelA protein could, indeed, be efficiently manipulated with NIR laser irradiation. In addition, the sequence specificity of Apt was confirmed with little influence of PCH_2_-rDNA-UCNPs on the natural trafficking behavior of RelA under TNF*α* stimulation, irrespective of NIR laser irradiation (Supplementary Fig. 13). Besides, a control aptamer-modified UCNPs (termed PCH_2_-Apt-UCNPs-control), where the cDNA of PCH_2_-Apt-UCNPs was replaced with a control sequence that cannot hybridize to the aptamer (Supplementary Table 1), was tested. Even with NIR laser irradiation, this control nanoparticle was unable to mediate the nuclear translocation of RelA (Supplementary Fig. 10), suggesting that protein regulation was originated from the conformation switch of aptamer. The capability of PCH_2_-Apt-UCNPs for manipulating the subcellular localization of native RelA protein was further confirmed with Western blot assays (Fig. 4d).

### Optical regulation of target protein-related genetic program

**Fig. 5:**
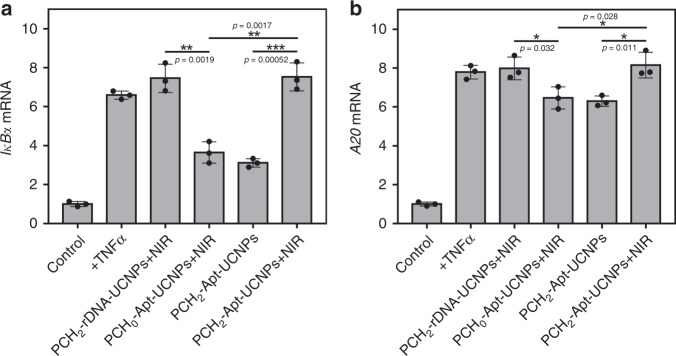
NIR-responsive manipulation of native RelA localization-related genetic program. Q-RT-PCR analysis of *IκBα* mRNA expression **a** and *A20* mRNA expression **b** after A549 cells were processed with different treatments. Except for the control (untreated) sample, all other samples were stimulated with TNFα. NIR laser irradiation time was fixed at 30 min. The experimental data were normalized using the GAPDH as an internal reference. Relative expression of these two mRNAs was calculated by setting the corresponding value of the control sample as 1. All data were collected from three independent experiments and presented as mean values ± S.D. *P ≤ 0.05, **P ≤ 0.01, ***P ≤ 0.001 by two-tailed unpaired Student’s *t*-test.

Correct subcellular localization of proteins is critical for their normal biofunction. We continued to test the potential of our aptamer-based photoresponsive nanoplatform for regulating RelA-related signaling pathways through manipulating its intracellular location. As reported, once translocation into nucleus, RelA could initiate a cascade of signaling pathways through alteration of internal genetic program (e.g., upregulation of *A20* and *IκBα*)^25,29^. A549 cells were processed with different treatments, and the RNAs were extracted to analyze the expression level of *A20* and *IκBα* with quantitative reverse transcriptase PCR (Q-RT-PCR). As shown in Fig. 5a, the expression level of *IκBα* mRNA was significantly enhanced after A549 cells were stimulated with TNFα. With treatment with PCH_2_-Apt-UCNPs, RelA was mainly retained in the cytoplasm, resulting in apparent suppression of *IκBα* mRNA. In comparison to the untreated group, the expression of *IκBα* mRNA was upregulated, to some extent, probably because that TNFα could stimulate multiple signaling pathways and the inhibition of RelA-related one could not completely block *IκBα*-associated genetic programs. Upon NIR laser irradiation, the expression of *IκBα* mRNA was restored to a level similar to that as the control sample with TNFα stimulation only, while no obvious change was observed in the sample of PCH_0_-Apt-UCNPs, indicating an important role of the PC linker-facilitated aptamer conformational switch for RelA activation. Meanwhile, neither PCH_2_-rDNA-UCNPs nor NIR laser irradiation treatments caused observable alteration in RelA-related *IκBα* program (Supplementary Fig. 14). Besides, the PCH_2_-Apt-UCNPs-control excluded the potential interference of bDNA fragments induced by the PC linker cleavage on cells. Similar to results of the expression assay of *IκBα* mRNA, only PCH_2_-Apt-UCNPs allowed photoregulation of *A20* mRNA expression (Fig. 5b and Supplementary Fig. 15). Collectively, these results demonstrated that, with the capability to manipulate the subcellular localization of specific protein in living cells, the current aptamer-based photoresponsive nanoplatform allowed regulation of target protein-related biological processes.

### Universality of this nanoplatform for protein regulation

To test the universality of this aptamer-based photoresponsive nanoplatform for protein regulation, another protein (lysozyme)-aptamer (LA, a lysozyme aptamer)^30^ system was designed (Supplementary Fig. 16). Our results showed that lysozyme could be effectively inhibited through aptamer-specific capture around the nanoparticle (Supplementary Fig. 17). On NIR irradiation, the captured lysozyme could be rapidly released to restore their catalytic activity. By using this PCH_2_-LA-UCNPs platform, NIR light-dependent activation of lysozyme for mediating bacterial viability was achieved in complex living *B. subtilis* system (Supplementary Fig. 18). These results demonstrated that the current nanoplatform could be potentially extended for photoregulation of different proteins in complex biological systems.

**Fig. 6:**
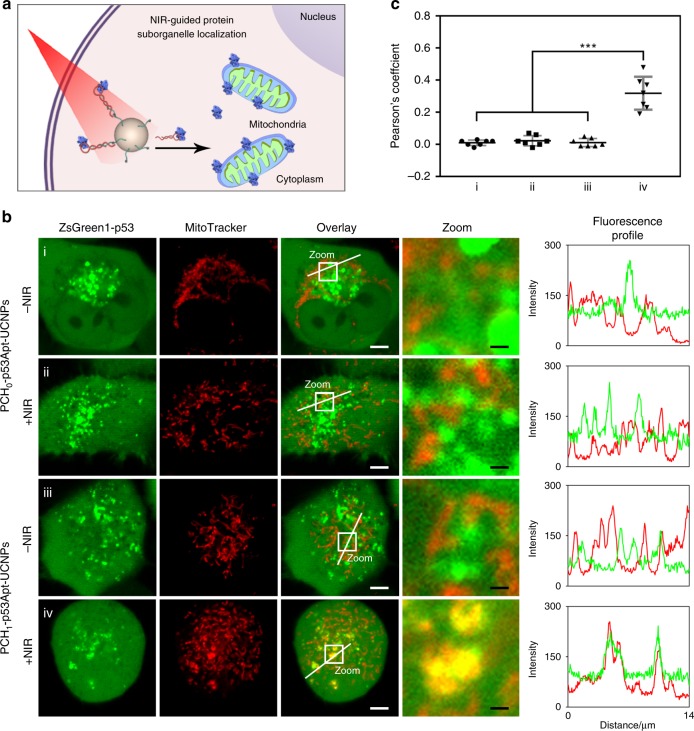
Manipulation of p53 protein localization in living H1299-ZsGreen1-p53R175H cells with NIR light irradiation. **a** Schematic illustration of the p53 transportation to mitochondria under NIR light irradiation. **b** CLSM imaging of living H1299-ZsGreen1-p53R175H cells pretreated with 100 μg mL^−1^ PCH_0_-p53Apt-UCNPs for 3 h and then irradiated with NIR laser for 0 min (i) or 10 min (ii), or pretreated with100 μg mL^−1^ PCH_1_-p53Apt-UCNPs for 3 h and then irradiated with NIR laser for 0 min (iii) or 10 min (iv). From left to right: fluorescence channel of ZsGreen1-p53, fluorescence channel of MitoTracker, overlay images, enlarged view of the zoom area in images, and the fluorescence profiling of ZsGreen1-p53/aptamer (green line) and MitoTracker (red line) in corresponding overlay images. Scale bars in overlay images and zoom images represent 5 μm and 0.65 μm, respectively. **c** Co-localization analysis of p53R175H/aptamer with mitochondria based on the corresponding seven CLSM images in (**b**). Seven images of co-localization analysis come from three independent experiments. The Pearson’s coefficient was calculated with an Image J software. Error bars represent the standard deviation of the Pearson’s coefficient data. Data are presented as mean values ± S.D. ***P  = 0.0000045 (i), 0.0000096 (ii) and 0.0000055 (iii) ≤0.001, by two-tailed unpaired Student’s *t*-test.

Next, we continued to test the potential of this aptamer-based nanoplatform for protein manipulation with different translocation modes, beyond cytoplasm-to-nucleus transfer, by setting up a p53/aptamer demonstration system. As reported, wild-type p53 could rapidly translocate from cytoplasm to mitochondria through binding with Bcl-2 family members to initiate the intrinsic apoptosis pathway^31^. In contrast, cancer-associated mutant p53 (p53R175H) failed to bind with Bcl-2 family members and lacked the capability to induce cell apoptosis, thus playing an active role in promoting cancer development and progression^32^. Binding of p53R175H with the specific aptamer was reported to potentially recuse its biological functions^33^. Herein, we used this aptamer to develop a photoresponsive nanoplatform (termed PCH_1_-p53Apt-UCNPs) for p53R175H regulation. A H1299 cell line with overexpression of ZsGreen1-fussed p53R175H was constructed. As visualized by CLSM, p53R175H was uniformly distributed all over the cells. With treatment of PCH_1_-p53Apt-UCNPs, bright fluorescence spots of p53R175H were observed and well colocalized with the nanoparticle signal, presumably resulting from specific protein capture by this aptamer-modified UCNPs (Supplementary Fig. 19). On irradiation with NIR light, the PC linker was cleaved to release the aptamer/p53R175H complex. Subsequently, an enhanced translocation of p53R175H into mitochondria was observed (Fig. 6). Whereas, compared with all the control samples, including PCH_1_-p53Apt-UCNPs without laser irradiation, PCH_0_-p53Apt-UCNPs with/without laser irradiation and the PCH_1_-p53Apt-control-UCNPs (where the aptamer was replaced with a scrambled RNA sequence) with/without laser irradiation (Supplementary Fig. 20), nearly 27-fold lower mitochondrial accumulation of p53R175H/aptamer could be detected. The capability of PCH_1_-p53Apt-UCNPs for NIR-responsive function recovery of p53R175H/aptamer was proved by the relatively high expression level of cleaved caspase 3, a critical indicator of p53-mediated apoptotic pathway, using western blot analysis (Supplementary Fig. 21). These results have preliminarily demonstrated that this aptamer-based photoresponsive nanoplatform could be extended for manipulating different proteins with different subcellular translocation modes.

## Discussion

In summary, we have developed an aptamer-based NIR light-responsive nanoplatform for manipulating the translocation activity of native proteins and thus regulating their subcellular localization with molecular specificity in living cells. Unlike conventional strategies, no chemical or genetic modification on target proteins was required in this design, and protein regulation was achieved through light-driven manipulation of the protein recognition unit, aptamer. Meanwhile, the limitation of commonly used UV light, e.g., poor biological penetration and high phototoxicity, were overcome by using UCNPs, which were capable of transforming excited NIR light into emitted UV light, as the core for specific protein assembly. Such efforts for minimizing artificial interference on the natural structure and expression of proteins would be essential for elucidating the potential roles that specific proteins play in complex biological networks. By using this nanoplatform, the subcellular translocation behavior of native RelA protein was precisely controlled in living cells with NIR light irradiation, enabling regulation of RelA-related signaling pathway in living cells.

Meanwhile, taking advantage of the modular design of this nanoplatform, another protein (p53R175H)/aptamer-UCNPs system has been successfully built. Its capability for NIR-responsive control over the subcellular translocation of p53R175H from cytoplasm to mitochondria was preliminarily proved. With the rapid advance of aptamers and nucleic acid chemistry, the current platform could be extended for manipulation of different bio-molecules with different subcellular translocation modes. On the other hand, as limited by the relatively large size of current UCNPs (around 50–100 nm in diameter), it is rather challenging to implement reversible control over the protein translocation from small organelles (e.g., nucleus and mitochondria) to cytoplasm. Meanwhile, it is still challenging to achieve guidance of target proteins to a desired location using the current system. Further efforts are needed to study the potential impact of this aptamer-based nanoplatform on the cellular status and to enhance its capacity for more precise and subtle protein manipulation. Also, for practical applications, the potential off-target effects of this nanoplatform needs to be further evaluated.

## Methods

### Materials and reagents

DNA synthesis reagents were purchased from Glen Research (Sterling, VA). NaCl, Hochest 33258, HEPES buffer and lysozyme were obtained from Sigma-Aldrich (Shanghai, China). Gd(CH_3_CO_2_)_3_•xH_2_O (99.9%), Y(CH_3_CO_2_)_3_•xH_2_O (99.9%), Yb(CH_3_CO_2_)_3_•4H_2_O (99.9%), Tm(CH_3_CO_2_)_3_•xH_2_O (99.9%), NaOH (98 ≥ %), NH_4_F (98 ≥ %), 1-octadecene (ODE), oleic acid (OA), and 2-aminoethyl dihydrogenphosphate (AEP) were purchased from J&K Scientific Ltd. Triton X-100, BSA (Fraction V), nuclear or cytoplasmic protein extraction kit and CCK-8 were obtained from Beyotime Institute of Biotechnology (China). Phosphate-buffered Saline (PBS) 10× (pH 7.4) and LysoTracker Green were obtained from Life Technologies. Tris-(2-carboxyethyl)-phosphine hydrochloride (TCEP) was purchased from Sangon Biotech (Shanghai, China). Sulfo-SMCC was obtained from Tokyo Chemical Industry (Shanghai, China). Paraformaldehyde (4%) was obtained from Beijing DingGuoChangSheng Biotechnology (Beijing, China). Human TNF-α was obtained from Pepro Technology (Suzhou, China). DAPI was obtained from Genview. Eastep® Super Total RNA Extraction Kit was obtained from Promega. FastQuant RT Kit (with gDNase) was obtained from TianGen Biotechnology (Beijing, China). 2×SYBR Green qPCR Master Mix (Low ROX) was obtained from Bimake (Shanghai, China). RPMI-1640 culture medium and DPBA (Gibco) were obtained from Thermo Fisher Scientific (Shanghai, China). Milli-Q water (resistance >18 MΩ cm) was used to prepare all solutions. *B. subtilis* was obtained from the China General Microbiological Culture Collection Center (Beijing, China). MitoTracker Red CMXRos was purchased from the Dalian Meilun Biotechnology Co., LTD (Dalian, China).

### DNA synthesis and purification

Except for DNA template and primers obtained from Sangon Biotech (Shanghai, China), other DNA sequences (Supplementary Table 1) were synthesized on a PolyGen DNA synthesizer. Both the synthesis and deprotection procedures were performed according to the instructions provided by the reagents’ manufacturers. The final DNA products were desalted with illustra NAP-5 columns (GE Healthcare), and the concentration was determined by detecting the UV absorption at 260 nm on the BioSpec-nano (SHIMADZU).

### Synthesis of NaGdF_4_:Yb/Tm (49.5/0.5 mol%) Core

Hexagonal phase NaGdF_4_:Yb/Tm nanoparticles were synthesized by thermal decomposition of trifluoroacetate precursors^34^. In detail, a 2 mL water solution of Ln(CH_3_CO_2_)_3_ (0.2 M, Ln = Gd, Yb, and Tm) was added to a 50 mL flask containing 4 mL of oleic acid. The mixture was then heated at 150 °C for 30 min to completely remove water from the solution. Then, 6 mL of 1-octadecene were quickly added to the flask, and the resulting mixture was heated at 150 °C for another 30 min before cooling down to 50 °C. Shortly thereafter, 5 mL of methanol solution containing NH_4_F (1.36 mmol) and NaOH (1 mmol) were added, and the resultant solution was stirred for 30 min. After evaporation of methanol, the solution was heated to 290 °C under argon for 1.5 h and then cooled down to room temperature. Ethanol (20 mL) was added and stirred for 10 min, collected by centrifugation, washed by ethanol and cyclohexane (*V*: *V* = 1:1), and then dispersed in 5 mL cyclohexane before further use.

### Synthesis of NaGdF_4_:Yb/Tm@NaYF_4_

The NaYF_4_ shell precursor was first prepared by mixing a 2 mL water solution of Y(CH_3_CO_2_)_3_ (0.1 M) and 4 mL oleic acid in a 50 mL flask followed by heating at 150 °C for 30 min. Then, 6 mL of 1-octadecene were added, and the mixture was heated at 150 °C for another 30 min before cooling down to 50 °C. Subsequently, NaGdF_4_:Yb/Tm core (40 mg) dispersed in 2 mL cyclohexane was added, along with a 5 mL methanol solution of NH_4_F (1.36 mmol) and NaOH (1 mmol). The resulting mixture was stirred at 50 °C for 30 min. After that, the solution was heated to 290 °C under argon for 1.5 h and then cooled down to room temperature. Ethanol (20 mL) was added and stirred for 10 min, collected by centrifugation, washed by ethanol and cyclohexane (*V*: *V* = 1:1), and then dispersed in 5 mL cyclohexane before further treatment.

### Synthesis of upconversion nanoparticles (UCNPs)

The as-prepared oleic acid-capped nanoparticles were dispersed in a mixed solution of ethanol (1 mL) and HCl (0.2 M, 1 mL)^35^. The mixture was sonicated for 5 min and collected by centrifugation. Subsequently, the resulting products were washed with ethanol/H2O (*V*: *V* = 1:1) three times and redispersed in H_2_O to form 2 wt% ligand-free nanoparticles. AEP (200 mg) was first dispersed in 10 mL water, and then ligand-free nanoparticles (2 mL, 2 wt%) were added and stirred vigorously over 48 h at room temperature. After that, AEP-stabilized UCNPs were collected by centrifugation at 12,000 rpm for 5 min, washed with deionized water, and redispersed in deionized water.

### DNA modification of UCNPs

Thiolated DNAs were modified onto AEP-stabilized UCNPs by amino-thiol reaction using a sulfo-SMCC crosslinker. First, the disulfide bond of thiolated DNAs was cleaved by treatment with TCEP (50×) at pH 5.0 for 1 h, and then the DNAs were desalted with illustra NAP-5 columns. Subsequently, 1 mg AEP-stabilized UCNPs was dissolved in 800 μL 10 mM HEPES buffer, and the resulting mixture was ultrasonicated for 30 min. Meanwhile, 0.4 mg sulfo-SMCC was dissolved in 200 μL 10 mM HEPES buffer through ultrasonication and then mixed fully with the solution of 1 mg AEP-stabilized UCNPs. After mixing at 25 °C for 2 h, the amino-activated UCNPs were collected by centrifugation at 12,000 rpm for 5 min, washed with 10 mM HEPES buffer three times, and redispersed in 200 μL 10 mM HEPES buffer. Then, 20 μL 100 μM thiolated DNA and 780 μL 10 mM HEPES buffer were added into the prepared UCNPs solution to reach a final volume of 1 mL, followed by mixing at 25 °C in dark overnight. Finally, the obtained DNA-modified UCNPs were collected by centrifugation at 12000 rpm for 5 min. After being washed three times with 1× PBS buffer, the resultant products were redispersed in 1× PBS buffer for further experimentation. A UV-2450 spectrophotometer (Shimadzu) was used to measure the absorption of the DNA-modified UCNPs. The hydrodynamic diameter and zeta potential of DNA-modified UCNPs was measured using a Zetasizer Nano ZS90 DLS system (Malvern Instruments Ltd., Worcestershire, England).

### Fluorescence measurements

UV light at 365 nm was provided by an Ultraviolet Analyzer (Hangzhou Qiwei Co., Ltd.), and near-infrared light at 980 nm was provided by a 980 nm Infrared Diode Laser (MDL-H-980-4W). The distance between the samples and the UV or near-infrared light source was fixed at 1 cm. To evaluate the light-driven strand displacement reaction, the cDNA and the aptamer were modified with a Cy3 fluorophore and a Cy5 fluorophore, respectively. Generally, 150 μL 400 nM DNA solutions or 150 μL 500 μg mL^−1^ DNA-modified UCNPs samples were irradiated with a UV light (24 W) or a 980 nm light (2 W) for a certain time (0 min, 0.5 min, 1 min, 5 min, 10 min, 20 min, 30 min, and 60 min), and the fluorescence spectra were recorded under excitation at 543 nm using a Fluoromax-4 spectrofluorometer (HORIBA JobinYvon, Edison, NJ).

### Cell culture and viability test

A549 cells were used in all cell experiments and cultured in RPMI 1640 medium supplemented with 10% Fetal Bovine Serum (FBS) in a 5% CO_2_, 37 °C incubator. A549 cells were seeded in a 96-well plate at a density of 5000 cells/well. After 24 h incubation, cells were treated with DNA-UCNPs of different concentrations for a certain time. After irradiation with 980 nm NIR laser (3 W cm^−2^, 4 min break after 2 min irradiation) for a certain time (0 min, 10 min, 20 min, and 30 min), cells were washed three times and cultured for another 16 h in fresh medium. Finally, the CCK-8 assay was performed, and the absorbance at 450 nm was measured with a Synergy 2 microplate reader (Gene Co., Ltd.). Cell viability was calculated as described by the manufacturer.

### Cellular uptake and intracellular distribution study

To study the cellular uptake and intracellular distribution of DNA-modified UCNPs, A549 cells were seeded in a 35-mm confocal dish and then incubated with RPMI-1640 culture medium containing 10% FBS and 100 μg mL^−1^ DNA-modified UCNPs for a certain time. The cells were washed three times with DPBS buffer and further stained with 2 μg mL^−1^ Hochest 33258 and 100 nM LysoTracker Green for another 20 min, followed by imaging with a FV1000 confocal microscope (Olympus). The adjusted settings of the instrument were listed as below: Laser 1 Wavelength (405 nm), Transmissivity (20.0%), PMT Voltage (904 V), LUT values of images (Min 564 and Max 3890); Laser 2 Wavelength (488 nm), Transmissivity (20.0%), PMT Voltage (725 V), LUT values of images (Min 558 and Max 3536); Laser 3 Wavelength (635 nm), Transmissivity (20.0%), PMT Voltage (793 V), LUT values of images (Min 446 and Max 3964). Early endosomal localization of DNA-modified UCNPs was also study via immunostaining. Briefly, the sample treated cells were fixed in 4% paraformaldehyde for 20 min and permeabilized with 0.2% Triton in PBS for 30 min, followed by blocking with 5% BSA 1 h at room temperature. Then, the cells were labeled with EEA1 (C45B10) Rabbit mAb (1:1000, Cell Signaling Technology, Cat:3288T) at 4 °C overnight. After 18 h, the cells were washed three times with DPBS and stained with secondary antibody (goat anti-rabbit) labeled with Alexa Fluor®594 (1:500, ImmunoReagents, Cat: IR2193) for 1 h at room temperature. After that, the cells were stained with DAPI for 20 min and then imaged using a ×60 oil objective of the inverted microscope (Nikon Ti-E, Nikon, Japan). All the settings were listed as below: for DAPI-Emission wavelength (450.0 nm), excitation wavelength (405.4 nm), pinhole radius (91.95), laser power (5.00%), high voltage (95) and AutoLUT setting; for mCherry-emission wavelength (595.0 nm), excitation wavelength (560.7 nm), pinhole radius (91.95), laser power (15.37%), high voltage (72) and AutoLUT setting; for Cy5-emission wavelength (700.0 nm), excitation wavelength (639.8 nm), pinhole radius (91.95), laser power (2%), high voltage (50) and AutoLUT setting.

### Immunostaining and fluorescence imaging assays

A549 cells were previously seeded in a 35-mm confocal dish and incubated with RPMI-1640 culture medium containing 10% FBS and 100 μg mL^−1^ DNA-modified UCNPs for a certain time. The cells were then stimulated with 2.5 ng mL^−1^ TNF-α at 37 °C for 1.5 h. After washing three times with DPBS (5 min each), the cells were fixed in 4% paraformaldehyde for 20 min and permeabilized with 0.2% Triton in PBS for 30 min, followed by blocking with 5% BSA 1 h at room temperature. Then, the cells were labeled with *NF-κβ* P65 (RelA) primary antibody (rabbit polyclonal, 1:500, Santa Cruz Biotechnology, Cat:sc-372) at 4 °C overnight. After 18 h, the cells were washed three times with DPBS and stained with secondary antibody (goat anti-rabbit) labeled with Alexa Fluor®594 (1:500, ImmunoReagents, Cat: IR2193) for 1 h at room temperature. After that, the cells were stained with DAPI for 20 min and then imaged with an oil dipping objective on the Nikon TI-E+A1 SI confocal laser scanning microscope (Japan). All the settings were listed as below: for DAPI-emission wavelength (450.0 nm), Excitation Wavelength (405.4 nm), pinhole radius (91.95), laser power (5.00%), high voltage (84) and AutoLUT setting; for mCherry-emission wavelength (595.0 nm), excitation wavelength (560.7 nm), pinhole radius (91.95), laser power (15.37%), high voltage (72) and AutoLUT setting.

### Western blot analysis

A549 cells were processed with different treatments by following the procedure described above. Cytoplasmic proteins were then collected using a cytoplasmic protein extraction kit (Beyotime Institute of Biotechnology, China). The harvested proteins were fractionated by 10% SDS-PAGE, electro-transferred to a polyvinylidene difluoride (PVDF) membrane, blocked with 10% BSA and incubated with NFkB P65 (RelA) primary antibody (rabbit polyclonal, 1:500, Santa Cruz Biotechnology, Cat:sc-372) or Tubulin alpha Antibody (1:1000, Absin Bioscience, Cat:abs130396) at 4 °C overnight. After being washed, the resulting PVDF membranes were stained with Goat anti-Rabbit IgG labeled with Peroxidase (1:5000, EMD Millipore Corporation, Cat:AP132P) and visualized using ECL Western blotting detection reagents. H1299-ZsGreen1-p53R175H cells were processed with different treatments according to a similar experimental protocol as described above, except that an antibody of Cleaved Caspase-3 (Asp175) (1:1000, Cell Signaling Technology, Cat:9661T) was applied. The fluorescein signal of gels was collected with the molecular imager (BIO-RAD). All image data were analyzed with the Image Lab^TM^ Software.

### Quantitative reverse transcriptase PCR (Q-RT-PCR)

A549 cells were processed with different treatments by following the procedure described above. Total RNA was then extracted using Eastep® Super Total RNA Extraction Kit. Then, 2 μg of RNA were reverse transcribed using FastQuant RT Kit (With gDNase) in a 20 μL reaction mixture. The resulting cDNA product was amplified in a 20 μL reaction mixture containing 10 μL of 2×SYBR Green qPCR Master Mix (2×SYBR Green qPCR Master Mix (Low ROX)) and 0.4 μM each of forward and reverse gene-specific primers. Samples were denatured for 15 min at 95 °C and then subjected to 40 cycles of 15 s at 94 °C, 34 s at 50 °C, and 34 s at 72 °C in an Applied Biosystems 7500 Real-Time System. Gene expression was calculated with the *ΔΔ*Ct method using untreated cells as a reference.

### Real-time monitoring of lysozyme activity

Lysozyme can catalyze the hydrolysis of micrococcus lysodeikticus (Huich Bio-tech Inc.), resulting in decreased absorbance at 450 nm (A450). Briefly, lysozyme (80 U mg^−1^ mL^−1^) were pretreated with PBS (as a blank control), lysozyme aptamer, UCNPs only, LA-UCNPs, or rDNA40-UCNPs. The resultant mixture were added into the micrococcus lysodeikticus solution (A450 = 0.7, volume = 400 μL), and the kinetic absorbance spectra were immediately recorded using a UV-2450 spectrophotometer (Shimadzu).

### Imaging of live and dead *B. subtilis*

Living *B. subtilis* was stained with the LIVE/DEAD BacLight Bacterial Viability Kits (Invitrogen) based on the instructions provided by the reagents’ manufacturers. To test the capability of PCH_2_-LA-UCNPs for manipulating the lysozyme activity in living *B. subtilis* system, PCH_2_-LA-UCNPs were mixed with active lysozyme for 10 min. The mixture was incubated with living *B. subtilis*. The resultant *B. subtilis* samples were irradiated with 980 nm NIR laser (3 W cm^−2^, 4 min break after 2 min irradiation) for a certain time (0 min and 10 min) and subsequently imaged with the LSM 880 with Airyscan confocal laser scanning microscope (Carl Zeiss GmbH, Jena, Germany). The adjusted settings of the instrument were listed as below: PI channel Wavelength (543 nm), laser power (2.0%), pinhole (90.1), gain (600); SYTO 9 channel wavelength (488 nm), laser power (5.0%), pinhole (90.1), gain (600), T PMT gain (380). The intensity minimum to black and intensity maximum to white value of images were 32 and 225, respectively.

### Synthesis of p53 RNA aptamer

Briefly, DNA templates for transcription were first amplified with PCR using the primers listed in Supplementary Table 1. The p53 RNA aptamer was synthesized by in vitro transcription with the DuraScribe T7 Transcription Kit by following the manufacture’s instruction.

### Imaging of p53 protein in living cells

To study p53 protein co-localization with mitochondria in living H1299-ZsGreen1-p53R175H cells, cells were processed with 100 μg mL^−1^ PCH_0_-Apt-UCNPs/PCH_1_-Apt-UCNPs at 37 °C for 3 h. After washing three times with DPBS, cells were stained with the MitoTracker Red CMXRos at 37 °C for 15 min. After removing unstained dyes with fresh medium, cells were irradiation with 980 nm NIR laser (3 W cm^−2^, 4 min break after 2 min irradiation) for a certain time (0 min and 10 min) and subsequently imaged with the LSM 880 with Airyscan confocal laser scanning microscope (Carl Zeiss GmbH, Jena, Germany). The adjusted settings of the instrument were listed as below: ZsGreen1 channel Wavelength (488 nm), Laser Power (10.0%), Pinhole (90.1), gain (600); MitoTracker Red channel wavelength (543 nm), laser power (2.0%), pinhole (90.1), gain (520); Cy5 channel wavelength (633 nm), laser power (10.0%), pinhole (90.1), gain (600). The intensity minimum to black and intensity maximum to white value of images were 0 and 225, respectively.

### Statistics and reproducibility

All numerical data, TEM imaging data, fluorescence imaging data of A549 cells and fluorescence imaging data of living H1299-ZsGreen1-p53R175H cells are collected from a minimum of three independent experiments unless otherwise specified. No data were excluded in the studies. Numerical data are presented as mean values ± S.D. Two-tailed unpaired Student’s *t*-test is used to assess significance (*P*-value). Fluorescence spectral data and UV absorbance data were analyzed with the OriginPro 9.0 (version 9.0) and the GraphPad Prism 7 (version 7.0). Statistical mean and differences were evaluated using Microsoft excel 2013’s statistical tools and the GraphPad Prism 7 (version 7.0). The gel image data were analyzed with the Image LabTM Software (version 6.0) and Image J (version 1.80). Confocal imaging data were analyzed using the Nikon Analysis Software (Nikon TI-E+A1 SI), Carl Zeiss ZEN 2 (blue edition) and Image J (version 1.80). Secondary structure and Gibbs free energy predictions of DNAs were collected from the NUPACK software (on-line analysis at www.nupack.org).

### Reporting summary

Further information on research design is available in the Nature Research Reporting Summary linked to this article.

## Supplementary information

Supplementary Information

Reporting Summary

## Data Availability

The main data in this work are available in the main manuscript and Supplementary Information. The source data underlying Figs. 2b–e, g, 3b, 4b–d, 5 and 6b, c and Supplementary Figs. 3–6, 8, 11, 12, 14, 15, 17, 18c and 21 are provided as a Source Data file. Additional data are available from the corresponding author upon reasonable request.

## Source data

Source Data
