# Supplementary material for: Aptamer-based optical manipulation of protein subcellular localization in cells
Source: Nat Commun. 2020 Mar 12;11:1347. doi: 10.1038/s41467-020-15113-2 (PMC7067792; doi:10.1038/s41467-020-15113-2)
Supplement: Supplementary file 1 — Supplementary Information [file 41467_2020_15113_MOESM1_ESM.pdf]

Supplementary Information

for

Aptamer-based Optical Manipulation of Protein Subcellular Localization in Cells

Xie *et al.*

Supplementary Figures

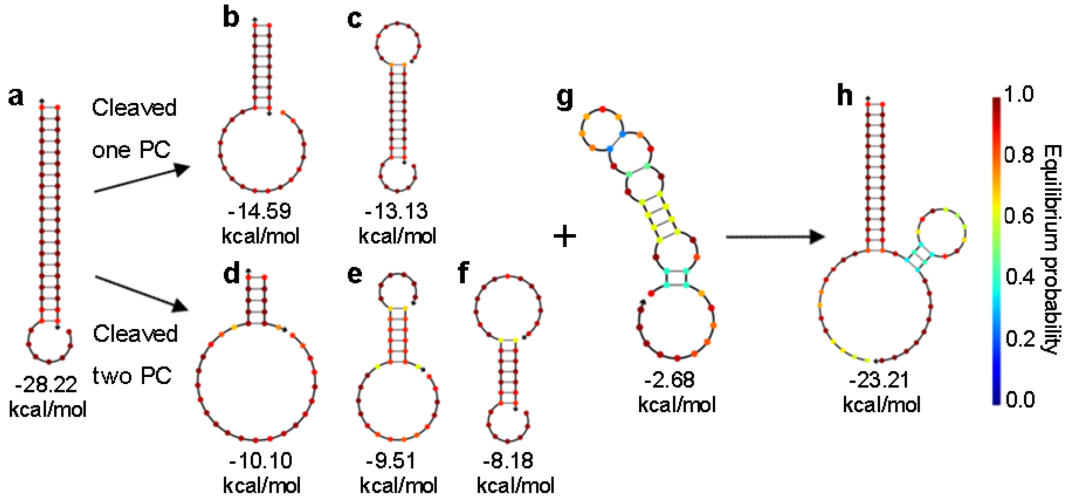

Supplementary Figure 1 | Simulation analysis. Secondary structure and Gibbs free energy (ΔG, -kcal mol^{-1}) predictions of the photocleavable DNA hybrids (a); the DNA fragments of photocleavable DNA hybrids (b-f); the RelA aptamer (g) and the DNA hybrids of RelA aptamer and its designed complementary DNA (h). The predictions are based on the NUPACK software analysis.

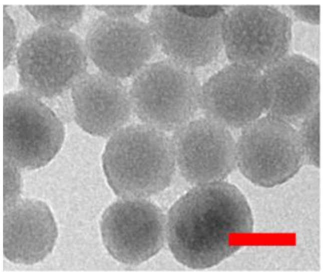

Supplementary Figure 2 | TEM analysis. TEM image of the UCNPs. The red scale bar represents 20 nm.

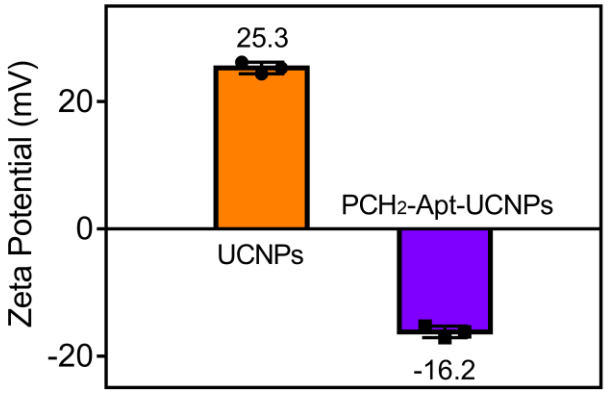

Supplementary Figure 3 | Potential analysis. Zeta potentials of the UCNPs and PCH_2-Apt-UCNPs. Error bars represent the standard deviation of three independent experiments. Data are presented as mean values ± S.D.

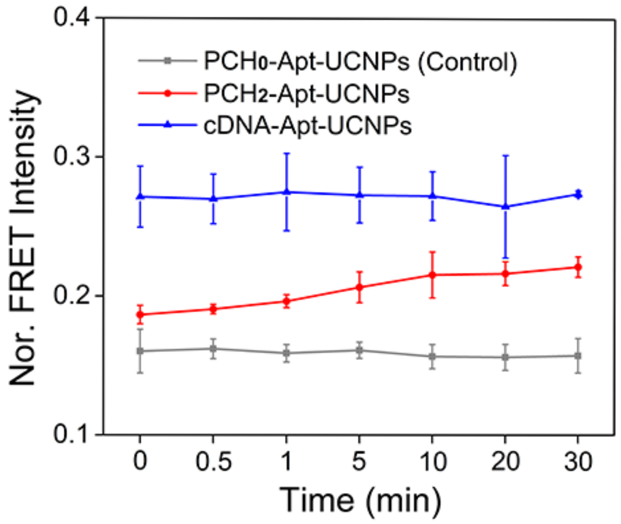

Supplementary Figure 4 | FRET analysis. Analysis of NIR irradiation time-dependent FRET changes for the nucleic acid replacement process of the PCH_2-Apt-UCNPs. Error bars represent the standard deviation of three independent experiments. Data are presented as mean values ± S.D.

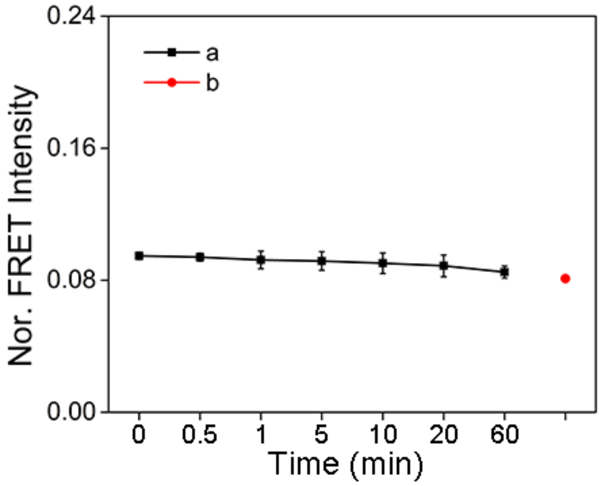

Supplementary Figure 5 | FRET analysis. Normalized FRET intensity of PCH_2-rDNA-UCNPs with (a) or without (b) a blocked DNA in response to NIR light irradiation for different lengths of time. Error bars represent the standard deviation of three independent experiments. Data are presented as mean values ± S.D.

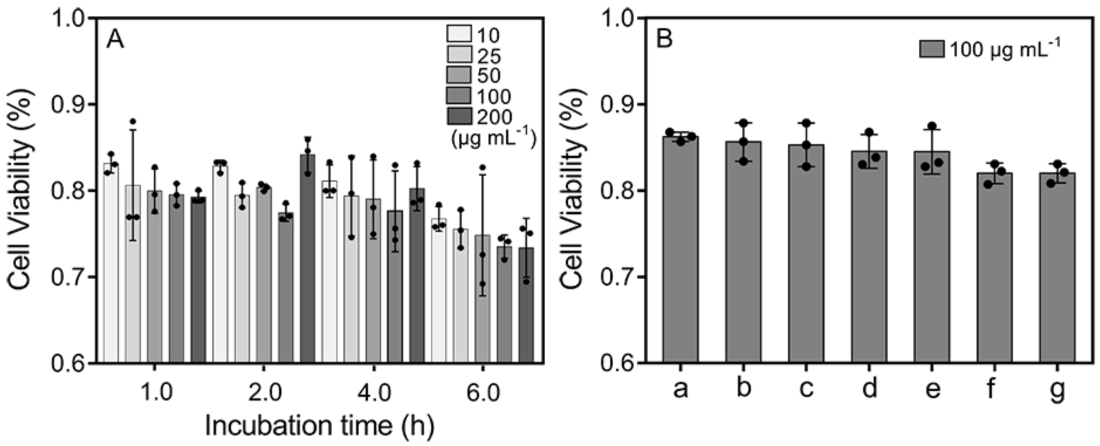

Supplementary Figure 6 | Cell viability. A549 cells incubated with (A) PCH_2-Apt-UCNPs (10, 25, 50, 100 and 200 μg mL^{-1}) for 1, 2, 4 and 6 h at 37 °C or (B) 100 μg mL^{-1} UCNPs (a); Apt-UCNPs (b); rDNA-UCNPs (c); PCH_2-Apt-UCNPs (d); PCH_2-rDNA-UCNPs (e); PCH_2-Apt-UCNPs irradiated with NIR 30 min (f) and PCH_2-rDNA-UCNPs irradiated with NIR 30 min (g) for 6 h at 37 °C. Error bars represent the standard deviation of three independent experiments. Data are presented as mean values ± S.D.

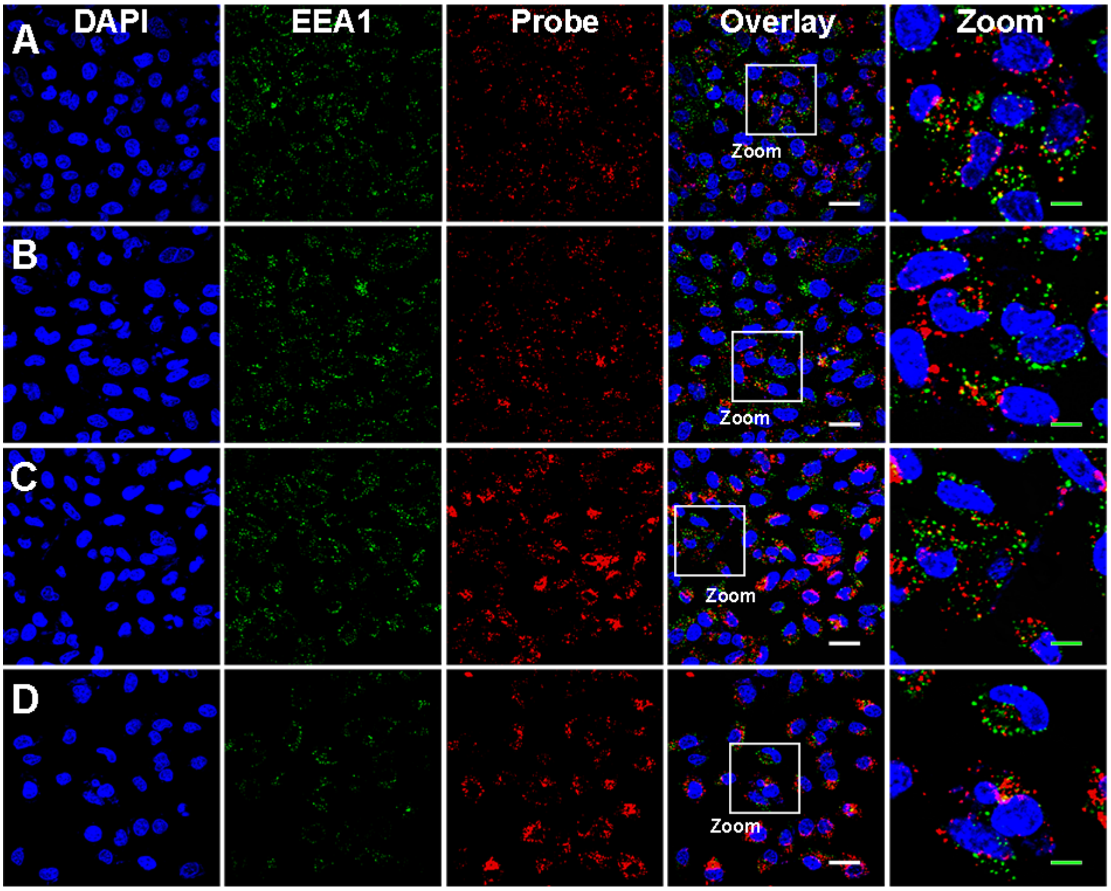

Supplementary Figure 7 | Fluorescence imaging analysis. Fluorescence images of A549 cells treated with 100 μg mL^{-1} PCH_2-Apt-UCNPs for 1 h (A), 2 h (B), 4 h (C) and 6 h (D). The PCH_2-Apt-UCNPs was traced with the Cy5 fluorescence signal. Nuclei and early endosomes were stained with DAPI (blue) and EEA1 (green), respectively. Scale bars in overlay images and zoom images represent 30 μm and 10 μm, respectively.

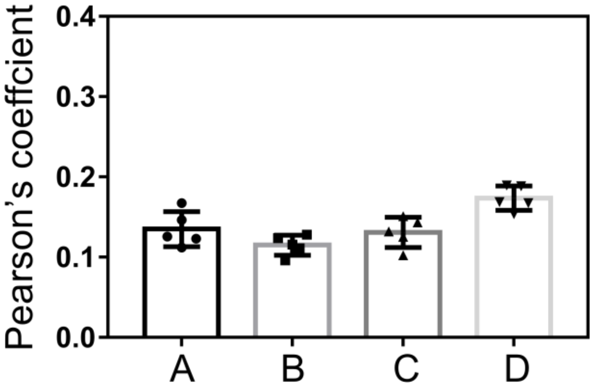

Supplementary Figure 8 | Co-localization analysis. Co-localization analysis of PCH_2-Apt-UCNPs with EEA1 based on the corresponding samples in Supplementary Figure 7. Five images of co-localization analysis come from three independent experiments. The Pearson’s coefficient was calculated with an ImageJ software. Error bars represent the standard deviation of the Pearson’s coefficient data. Data are presented as mean values ± S.D.

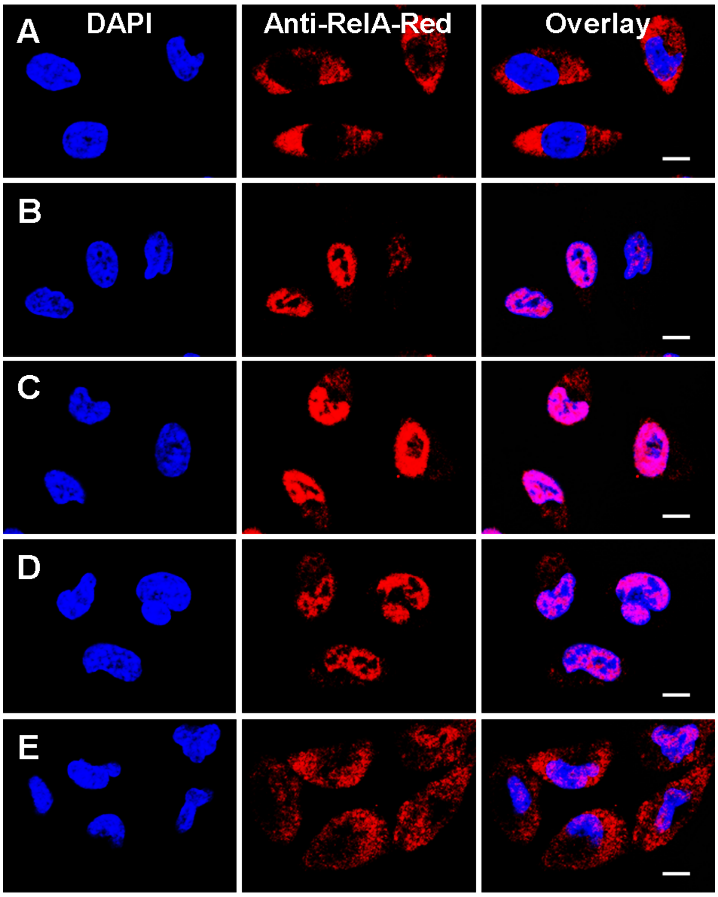

Supplementary Figure 9 | Fluorescence imaging analysis. Fluorescence images of A549 cells only (A); A549 cells stimulated with TNFα (2.5 ng mL^{-1}) for 1.5 h (B); A549 cells pretreated with 100 μg mL^{-1} UCNPs (C), PCH_2-rDNA-UCNPs (D) or PCH_2-Apt-UCNPs (E) and then stimulated with TNFα (2.5 ng mL^{-1}) for 1.5 h. Scale bars in overlay images represent 10 μm.

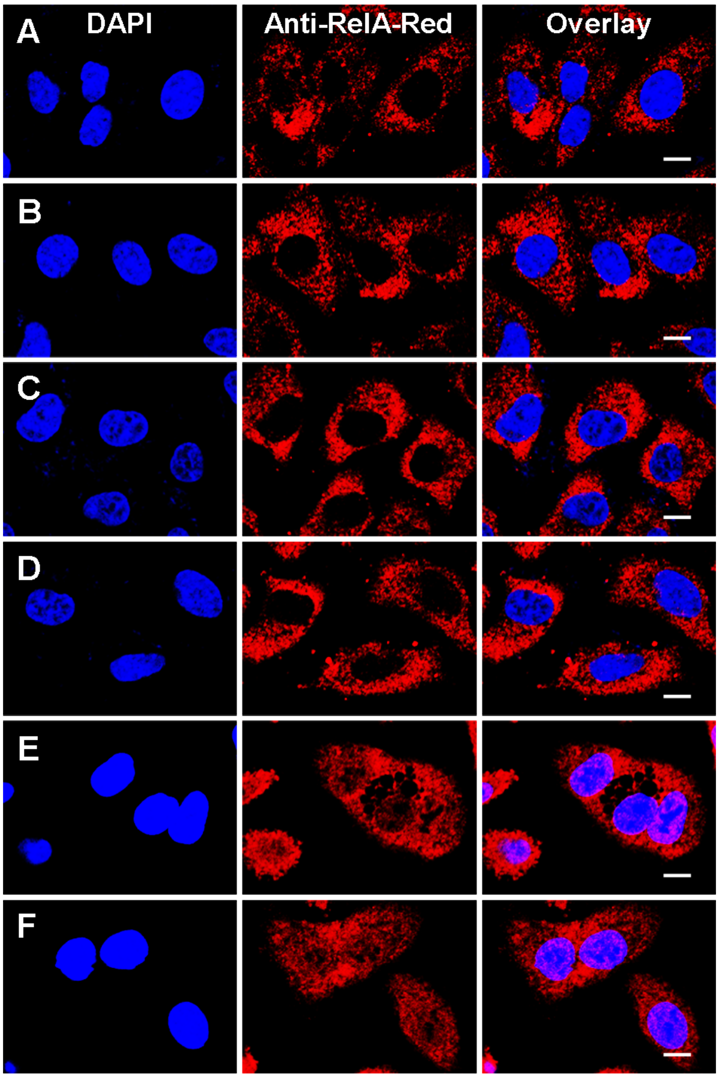

Supplementary Figure 10 | Fluorescence imaging analysis. Fluorescence images of A549 cells only (A), A549 cells irradiated with NIR for 30 min (B), A549 cells treated with 100 μg mL^{-1} UCNPs (C), A549 cells pretreated with 100 μg mL^{-1} UCNPs and then irradiated with NIR for 30 min (D), A549 cells pretreated with 100 μg mL^{-1} PCH_2-Apt-UCNPs-control, stimulated with TNFα (2.5 ng mL^{-1}) for 1.5 h, and then irradiated with NIR light for 0 min (E) or 30 min (F). Scale bars in overlay images represent 10 μm.

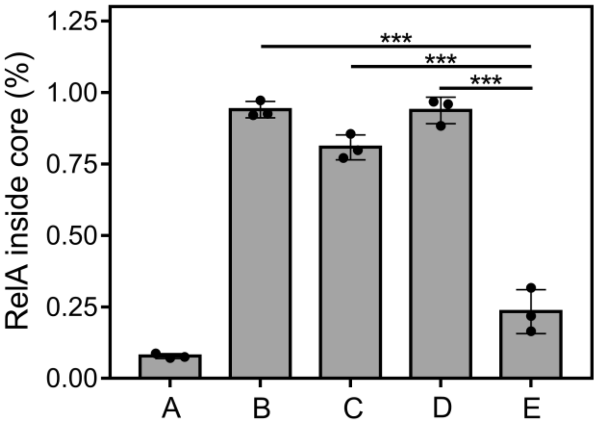

Supplementary Figure 11 | Fluorescence intensity analysis. Normalized fluorescence intensity of RelA protein in corresponding cell samples of Supplementary Figure 9. For the statistical analysis, data were obtained from three CLSM images and analyzed by using the Image J software for each condition. Error bars represent the standard deviation of three independent experiments. Data are presented as mean values ± S.D. ****P* = 0.00012 (B), 0.00035 (C) and 0.00017 (D) ≤ 0.001, by two-tailed unpaired Student's *t*-test.

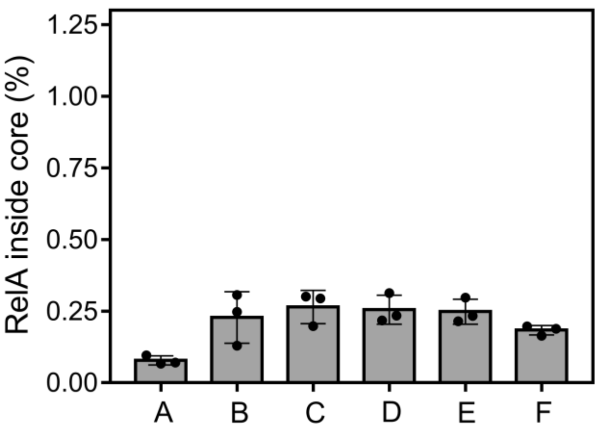

Supplementary Figure 12 | Fluorescence intensity analysis. Normalized fluorescence intensity of RelA protein in corresponding cell samples of Supplementary Figure 10. For the statistical analysis, data were obtained from three CLSM images and analyzed by using the Image J software for each condition. Error bars represent the standard deviation of three independent experiments. Data are presented as mean values ± S.D.

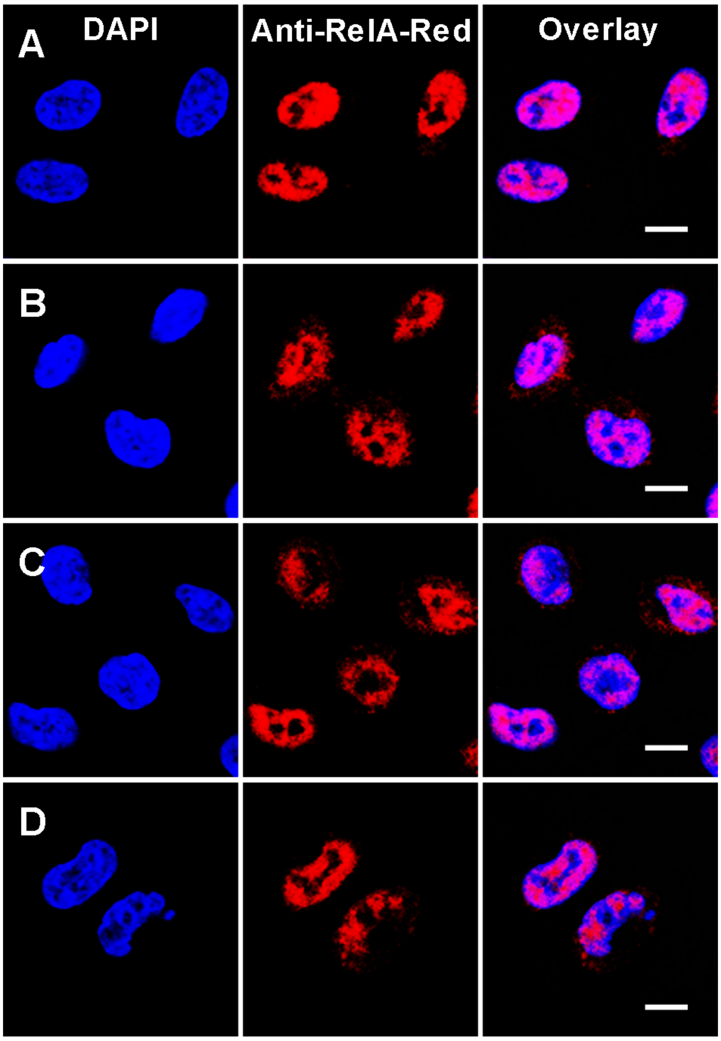

Supplementary Figure 13 | Fluorescence imaging analysis. Fluorescence images of A549 cells pretreated with 100 μg mL^{-1} PCH_2-rDNA-UCNPs, stimulated with TNF*α* (2.5 ng mL^{-1}) for 1.5 h, and then irradiated with NIR light for 0 min (A), 10 min (B), 20 min (C) or 30 min (D). Scale bars in overlay images represent 10 μm.

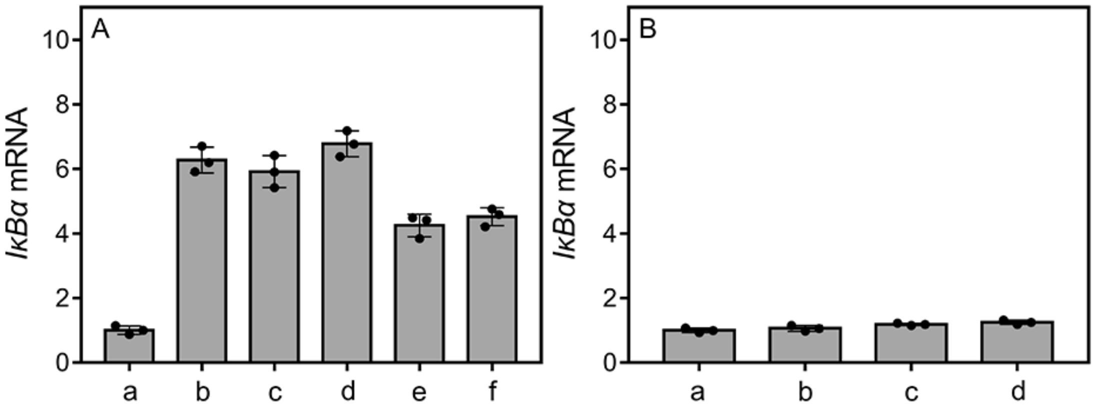

Supplementary Figure 14 | Q-RT-PCR analysis. (A) Fold change of gene expression in A549 cells only (a); A549 cells stimulated with TNF*α* (b); A549 cells pretreated with 100 μg mL^{-1} PCH_2-rDNA-UCNPs and then stimulated with TNF*α* (c), A549 cells stimulated with TNF*α* and then irradiated with NIR for 20 min (d), A549 cells pretreated with 100 μg mL^{-1} PCH_2-Apt-UCNPs-control, stimulated with TNF*α*, and then irradiated with NIR light for 0 min (e) or 30 min (f). (B) Fold change of gene expression in A549 cells only (a); A549 cells irradiated with NIR for 30 min (b); A549 cells treated with 100 μg mL^{-1} UCNPs (c), A549 cells pretreated with 100 μg mL^{-1} UCNPs and then irradiated with NIR for 30 min (d). Data were normalized to the GAPDH and expressed as fold change relative to A549 cells only (a). Error bars represent the standard deviation of three independent experiments. Data are presented as mean values ± S.D.

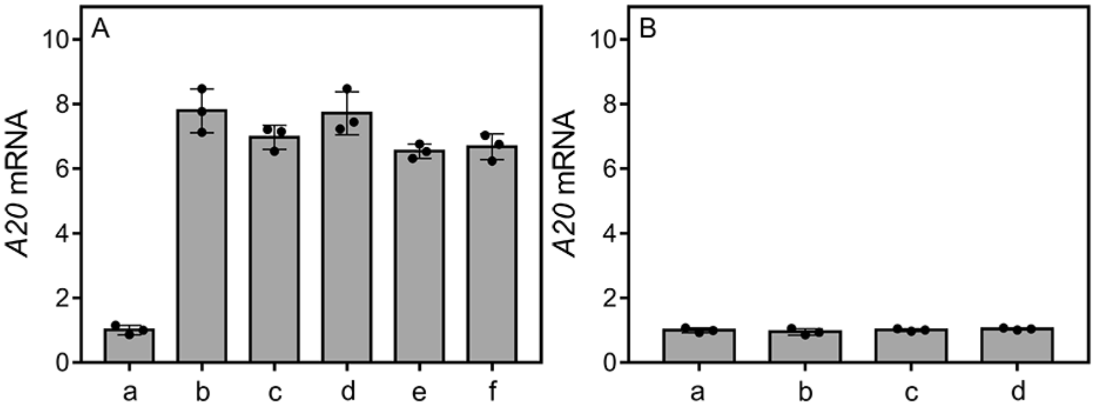

Supplementary Figure 15 | Q-RT-PCR analysis. (A) Fold change of gene expression in A549 cells only (a); A549 cells stimulated with TNF*α* (b); A549 cells pretreated with 100 μg mL^{-1} PCH_2-rDNA-UCNPs and then stimulated with TNF*α* (c), A549 cells pretreated with TNF*α* and then irradiated with NIR for 20 min (d), A549 cells pretreated with 100 μg mL^{-1} PCH_2-Apt-UCNPs-control, stimulated with TNF*α*, and then irradiated with NIR light for 0 min (e) or 30 min (f). (B) Fold change of gene expression in A549 cells only (a); A549 cells irradiated with NIR for 30 min (b); A549 cells treated with 100 μg mL^{-1} UCNPs (c), A549 cells pretreated with 100 μg mL^{-1} UCNPs and then irradiated with NIR for 30 min (d). Data were normalized to the GAPDH and expressed as fold change relative to A549 cells only (a). Error bars represent the standard deviation of three independent experiments. Data are presented as mean values ± S.D.

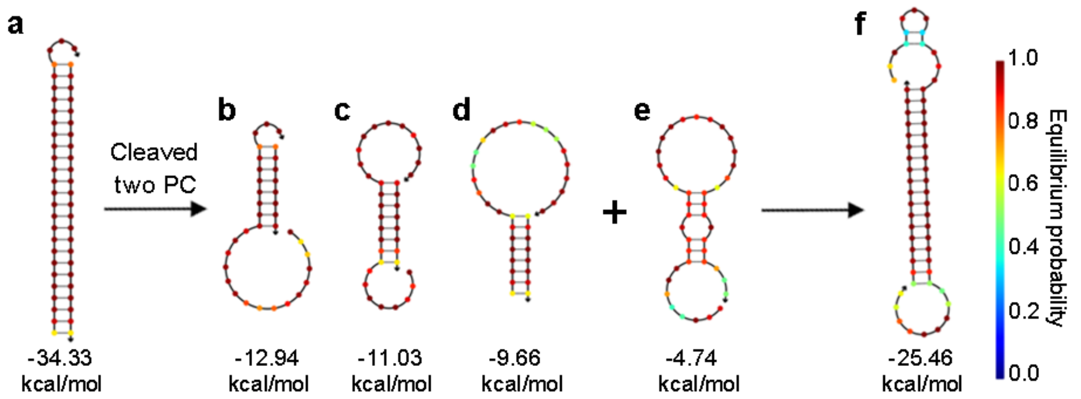

Supplementary Figure 16 | Simulation analysis. Secondary structure and Gibbs free energy (ΔG, -kcal mol^{-1}) predictions of the photocleavable DNA hybrids (a); the DNA fragments of photocleavable DNA hybrids (b-d); the lysozyme aptamer (e) and the DNA hybrids of lysozyme aptamer and its designed complementary DNA (f). The predictions are based on the NUPACK software analysis.

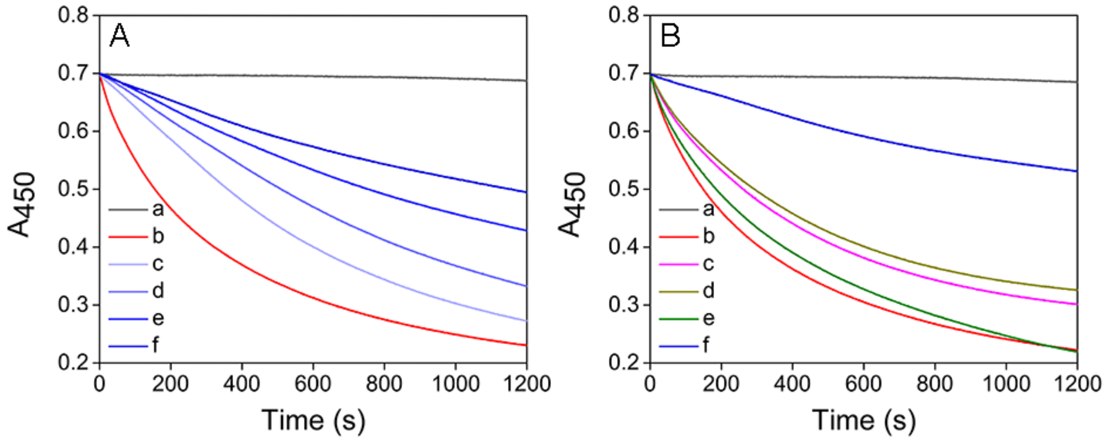

Supplementary Figure 17 | Kinetic spectra analysis. (A) Kinetic absorption spectra of the micrococcus lysodeikticus solution treated with reaction buffer (a); 80 U mg^{-1} mL^{-1} lysozyme (b); and 80 U mg^{-1} mL^{-1} lysozyme pretreated with LA-UCNPs of different concentrations: 10 μg mL^{-1} (c), 25 μg mL^{-1} (d), 50 μg mL^{-1} (e) and 100 μg mL^{-1} (f) LA-UCNPs. (B) Kinetic absorption spectra of the micrococcus lysodeikticus solution treated with reaction buffer (a); 80 U mg^{-1} mL^{-1} lysozyme (b); and 80 U mg^{-1} mL^{-1} lysozyme pretreated with 100 μg mL^{-1} UCNPs (c), 100 μg mL^{-1} $rDNA_{40}$-UCNPs (d), 400 nM lysozyme aptamer (e) or 100 μg mL^{-1} LA-UCNPs (f). The activity of lysozyme was evaluated by its capability for destroying the cell walls of micrococcus lysodeikticus, which could result in a decrease of absorbance at 450 nm.[1] The activity of lysozyme was efficiently inhibited by LA (lysozyme aptamer)-UCNPs in a concentration-dependent manner, while little inhibitory impact on lysozyme was caused by excess LA molecules, UCNPs or $rDNA_{40}$-UCNPs.

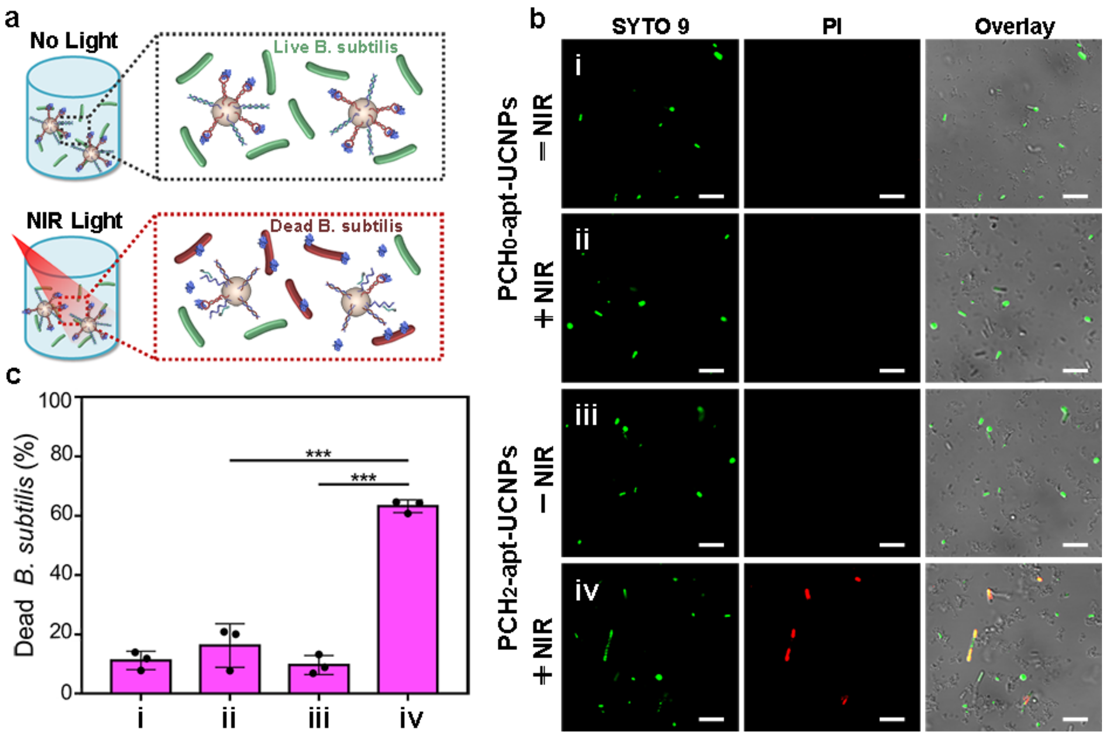

Supplementary Figure 18 | Optically Manipulating lysozyme activity in living *B. subtilis* system. (a) Schematic illustration of the aptamer-based NIR-responsive nanoplatform for manipulating the lysozyme activity in living *B. subtilis* system. (b) CLSM imaging of living *B. subtilis* with different treatments: PCH_0-LA-UCNPs-captured lysozymes irradiated by NIR laser for 0 min (i) and 10 min (ii); PCH_2-LA-UCNPs-captured lysozymes irradiated by NIR laser for 0 min (iii) and 10 min (iv). The concentration of lysozyme, and PCH-LA-UCNPs was 1000 U mg^{-1} mL^{-1} and 1 mg mL^{-1}, respectively. Scale bar represents 10 μm. (c) The percentage analysis of dead *B. subtilis* from Supplementary Figure 18b. All data were collected from three independent experiments and presented as mean values ± S.D. ****P* = 0.00044 (ii) and 0.000018 (iii) ≤ 0.001, by two-tailed unpaired Student's *t*-test.

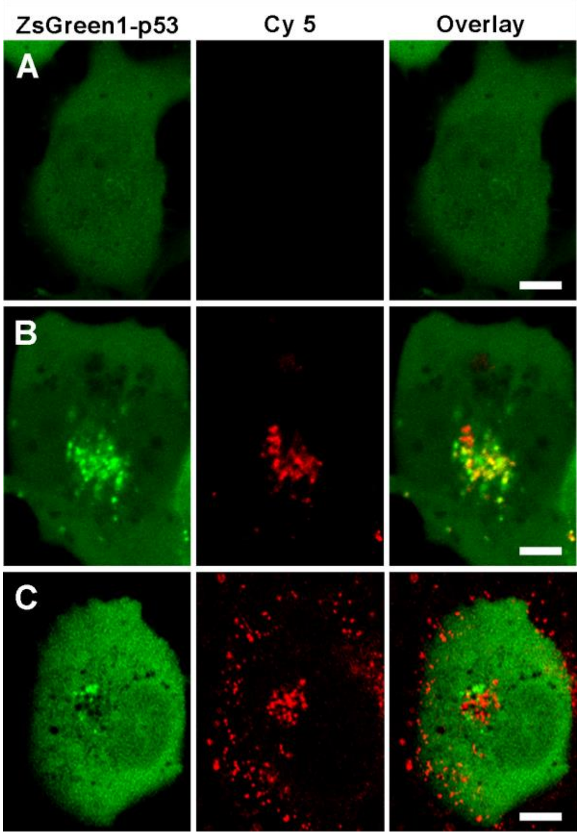

Supplementary Figure 19 | Fluorescence imaging analysis. CLSM imaging of living H1299-ZsGreen1-p53R175H cells without (A) and with (B) treatment of 100 μg mL^{-1} PCH_1-p53Apt-UCNPs for 3h and (C) treatment of 100 μg mL^{-1} PCH_1-p53Apt-control-UCNPs for 3h. The p53R175H protein was traced with the ZsGreen1 fluorescence signal. PCH_1-Apt-UCNPs was traced with the Cy5 fluorescence signal. Scale bars in overlay images represent 10 μm.

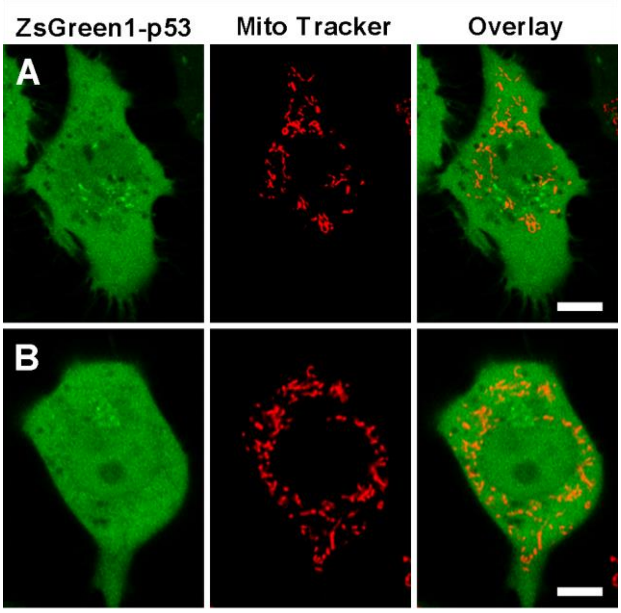

Supplementary Figure 20 | Fluorescence imaging analysis. CLSM imaging of living H1299-ZsGreen1-p53R175H cells pretreated with 100 μg mL^{-1} PCH_1-p53Apt-control-UCNPs for 3 h and then irradiated with NIR laser for 0 min (A) or 10 min (B). From left to right: fluorescence channel of ZsGreen1-p53, fluorescence channel of MitoTracker, overlay images. Scale bars in overlay images represent 10 μm.

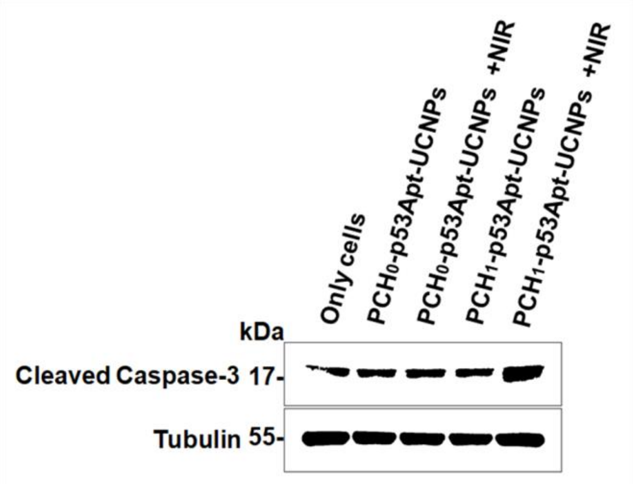

Supplementary Figure 21 | Western blot. Western blot analysis of cleaved caspase-3 in H1299-ZsGreen1-p53R175H cells pretreated with 100 μg mL^{-1} PCH_0- p53Apt-UCNPs for 3 h and then irradiated with NIR laser for 0 min or 10 min; and pretreated with100 μg mL^{-1} PCH_1-p53Apt-UCNPs for 3 h and then irradiated with NIR laser for 0 min or 10 min. The data is collected from a single experiment test.

Supplementary Table 1 | DNA sequences used in this study.

Name	Sequences (from 5' to 3') for RelA protein model
Cy5-RelA aptamer-SH	Cy5-GCG GGA CAG GAG AAA CAC GGC ATG TCA GCG TTT TTT TTT T-SH
RelA aptamer-SH	GCG GGA CAG GAG AAA CAC GGC ATG TCA GCG TTT TTT TTT T-SH
HS-PEG$_2$-cDNA-Cy3	HS-PEG$_2$-GAC ACT CCT CAA TTT CTC CTG TCC CGC-Cy3
HS-PEG$_2$-cDNA	HS-PEG$_2$-GAC ACT CCT CAA TTT CTC CTG TCC CGC
HS-PEG$_2$-cDNA-control	HS-PEG$_2$-AAA GCA ATT CGT CAA GTT AAT ACG ATG
Cy5-rDNA$_{30}$-SH	Cy5-NNN NNN NNN NNN NNN NNN NNN NNN NNN NNN TTT TTT TTT T-SH
rDNA$_{30}$-SH	NNN NNN NNN NNN NNN NNN NNN NNN NNN NNN TTT TTT TTT T-SH
bDNA-No PC	GCG GGA CAG GAG AAA TTG AG
bDNA-One PC	GCG GGA CAG-**PC**-GAG AAA TTG AG
bDNA-Two PC	GCG GGA-**PC**-CAG GAG A-**PC**-AA TTG AG
bDNA-Two PC-control	CAT CGT-**PC**-ATT AAC T -**PC**-TG ACG AA
GAPDH forward primer	ATG GGG AAG GTG AAG GTC G
GAPDH reverse primer	GGG GTC ATT GAT GGC AAC AAT A
IκBα forward primer	TAG CCT TCA GGA TGG AGT GG
IκBα reverse primer	TCC TGA GCT CCG AGA CTT TC
A20 forward primer	AAG CTG TGA AGA TAC GGG AGA
A20 reverse primer	CGA TGA GGG CTT TGT GGA TGA T
	Sequences (from 5' to 3') for lysozyme protein model
HS-lysozyme aptamer (LA)	HS-TTT TTT TTT TGC AGC TAA GCA GGC GGC TCA CAA AAC CAT TCG CAT GCG GC
cDNA-PEG$_2$-SH	GAA TGG TTT TGT GAG CCG CTC CTG TCC-PEG2-SH

HS-rDNA40	HS-T TTT TTT TTT NNN NNN NNN NNN NNN NNN NNN NNN NNN NNN NNN NNN NNN N
bDNA-No PC	CAG GAG CGG CTC ACA AAA CCA TTC
bDNA-Two PC	CAGGAGCG-PC-GCTCACAA-PC-AACCATTC
	Sequences (from 5’ to 3’) for p53 protein model
DNA template used for the transcription of p53 RNA aptamer in this work	GCT AGG TGA GTG GTG CTA ATA CGA CTC ACT ATA GGC AAT GGT ACG GTA CTT CCA TTA GCG CAT TTT AAC ATA GGG TGC CAA AAG TGC ACG CTA CTT TGT TTC GTT GTA ACG AAC GCG GC
Forward primer	GCT AGG TGA GTG GTG CTA ATA CG
Reverse primer	GCC GCG TTC GTT ACA ACG AAA C
p53 RNA aptamer (p53Apt)	GGC AAU GGU ACG GUA CUU CCA UUA GCG CAU UUU AAC AUA GGG UGC CAA AAG UGC ACG CUA CUU UGU UUC GUU GUA ACG AAC GCG GC
Scrambled RNA control (p53Apt-control)	GGC AAA UGU UGA UUA UGU UGU AUA CAG ACU CAG UGA UUG GCA ACC UAG GCC ACC AUG CUA CGC UGU UUC GUU GUA ACG AAC GCG GC
HS-cDNA-One PC-Cy5	HS-TTTTT-PC-TTTTT-Cy5-GCC GCG TTC GTT ACA ACG AAA
HS-cDNA-One PC	HS-TTTTT-PC-TTTTT-GCC GCG TTC GTT ACA ACG AAA
HS-cDNA-No PC	HS-TTTTT-TTTTT-GCC GCG TTC GTT ACA ACG AAA

Note: PEG refers to Spacer Phosphoramidite 18. **PC** refers to photocleavable (PC) linker, which is 3-(4,4'-Dimethoxytrityl)-1-(2-nitrophenyl)-propan-1-yl-[(2-cyanoethyl)-(N,N-diisopropyl)]-phosphoramidite (Catalog #:10-4920, Glen Research), as shown below. This photocleavable phosphoramidite can be incorporated into DNA strands that can be cleaved into DNA fragments under UV irradiation.

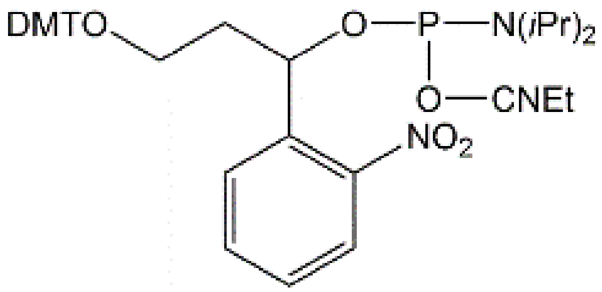

Supplementary Figure 22 | Photocleavable (PC) linker. The molecular structure of PC linker used in the Supplementary Table 1.

Supplementary References

1. Gorin, G., Wang, S.-F. & Papapavlou, L. Assay of lysozyme by its lytic action on M. lysodeikticus cells. *Anal. Biochem.* **39,** 113-127 (1971).
