# Supplementary material for: Aptamer-based optical manipulation of protein subcellular localization in cells
Source: Nat Commun. 2020 Mar 12;11:1347. doi: 10.1038/s41467-020-15113-2 (PMC7067792; doi:10.1038/s41467-020-15113-2)
Supplement: Supplementary file 2 — Reporting Summary [file 41467_2020_15113_MOESM2_ESM.pdf]

nature research

Corresponding author(s): Weihong Tan

Last updated by author(s): Feb 3, 2020

Reporting Summary

Nature Research wishes to improve the reproducibility of the work that we publish. This form provides structure for consistency and transparency in reporting. For further information on Nature Research policies, see Authors & Referees and the Editorial Policy Checklist.

Statistics

For all statistical analyses, confirm that the following items are present in the figure legend, table legend, main text, or Methods section.

n/a	Confirmed	
☐	☒	The exact sample size (*n*) for each experimental group/condition, given as a discrete number and unit of measurement
☐	☒	A statement on whether measurements were taken from distinct samples or whether the same sample was measured repeatedly
☐	☒	The statistical test(s) used AND whether they are one- or two-sided<br>*Only common tests should be described solely by name; describe more complex techniques in the Methods section.*
☐	☒	A description of all covariates tested
☒	☐	A description of any assumptions or corrections, such as tests of normality and adjustment for multiple comparisons
☐	☒	A full description of the statistical parameters including central tendency (e.g. means) or other basic estimates (e.g. regression coefficient) AND variation (e.g. standard deviation) or associated estimates of uncertainty (e.g. confidence intervals)
☐	☒	For null hypothesis testing, the test statistic (e.g. *F*, *t*, *r*) with confidence intervals, effect sizes, degrees of freedom and *P* value noted<br>*Give P values as exact values whenever suitable.*
☒	☐	For Bayesian analysis, information on the choice of priors and Markov chain Monte Carlo settings
☒	☐	For hierarchical and complex designs, identification of the appropriate level for tests and full reporting of outcomes
☐	☒	Estimates of effect sizes (e.g. Cohen's *d*, Pearson's *r*), indicating how they were calculated

Our web collection on statistics for biologists contains articles on many of the points above.

Software and code

Policy information about availability of computer code

Data collection	The quantitative analysis of oligonucleotides was performed on the BioSpec-nano (SHIMADZU). Fluorescence spectral data was measured on the Fluoromax-4 spectrofluorometer (HORIBA JobinYvon, Edison, NJ). The data of hydrodynamic diameter and zeta potential was measured using a Zetasizer Nano ZS90 DLS system (Malvern Instruments Ltd., Worcestershire, England). The CCK-8 assay data was measured with a Synergy 2 microplate reader (Gene Co., Ltd.). The gel imaging data was collected with the molecular imager (BIO-RAD). The UV absorption data was collected with a UV-2450 spectrophotometer (Shimadzu). The Q-RT-PCR data was collected on an Applied Biosystems 7500 Real-Time System. Confocal imaging data was collected with the LSM 880 with Airyscan confocal laser scanning microscope (Carl Zeiss GmbH, Jena, Germany) and the the Nikon TI-E+A1 SI confocal laser scanning microscope (Japan).
Data analysis	Fluorescence spectral data and UV absorbance data were analyzed with the OriginPro 9.0 (version 9.0) and the GraphPad Prism 7 (version 7.0). Statistical mean and differences were evaluated using Microsoft excel 2013's statistical tools and the GraphPad Prism 7 (version 7.0). The gel image data were analyzed with the Image LabTM Software (version 6.0) and Image J (version 1.80). Confocal imaging data was analyzed using the Nikon Analysis Software (Nikon TI-E+A1 SI), Carl Zeiss ZEN 2 (blue edition) and Image J (version 1.80). Secondary structure and Gibbs free energy predictions of DNAs were collected from the NUPACK software (on-line analysis at www.nupack.org)

For manuscripts utilizing custom algorithms or software that are central to the research but not yet described in published literature, software must be made available to editors/reviewers. We strongly encourage code deposition in a community repository (e.g. GitHub). See the Nature Research guidelines for submitting code & software for further information.

Data

Policy information about availability of data

All manuscripts must include a data availability statement. This statement should provide the following information, where applicable:

- Accession codes, unique identifiers, or web links for publicly available datasets
- A list of figures that have associated raw data
- A description of any restrictions on data availability

The main data in this work are available in the main manuscript and Supplementary Information. The source data underlying Figs 2b-e, 2g, 3b, 4b-d, 5 and 6b-c and Supplementary Figs 3, 4, 5, 6, 8, 11, 12, 14, 15, 17, 18c and 21 are provided as a Source Data file. Additional data are available from the corresponding author upon reasonable request.

Field-specific reporting

Please select the one below that is the best fit for your research. If you are not sure, read the appropriate sections before making your selection.

☒ Life sciences ☐ Behavioural & social sciences ☐ Ecological, evolutionary & environmental sciences

For a reference copy of the document with all sections, see nature.com/documents/nr-reporting-summary-flat.pdf

Life sciences study design

All studies must disclose on these points even when the disclosure is negative.

Sample size	Due to the the uncertainty of biological experiments, gene expression analysis and fluorescence imaging analysis involved were performed at least three independent times. Sample size (at least n=3) was chosen according to cited reference (Molecular & Cellular Proteomics 10.6 (2011)), which would allow for adequate analysis to make meaningful conclusion.
Data exclusions	No data was excluded from studies. And data significance is judged according to the P value.
Replication	Reproducibility of the data was confirmed by at least three independent experiments.
Randomization	Samples were randomly allocated into groups.
Blinding	Not applicable, as samples were processed with identical procedures that should not have bias outcomes.

Reporting for specific materials, systems and methods

We require information from authors about some types of materials, experimental systems and methods used in many studies. Here, indicate whether each material, system or method listed is relevant to your study. If you are not sure if a list item applies to your research, read the appropriate section before selecting a response.

Materials & experimental systems

n/a	Involved in the study	
☐	☒	Antibodies
☐	☒	Eukaryotic cell lines
☒	☐	Palaeontology
☒	☐	Animals and other organisms
☒	☐	Human research participants
☒	☐	Clinical data

Methods

n/a	Involved in the study	
☒	☐	ChIP-seq
☒	☐	Flow cytometry
☒	☐	MRI-based neuroimaging

Antibodies

Antibodies used	NF-κβ P65 (RelA) antibody (rabbit polyclonal) (1:500, Cat:sc-372) was obtained from Santa Cruz Biotechnology. goat anti-rabbit conjugated to Alexa Fluor®594 (1:500, Cat: IR2193) was obtained from ImmunoReagents. Tubulin alpha Antibody (1:1000, Cat:abs130396) was obtained from Absin Bioscience Inc. Goat anti-Rabbit IgG (Peroxidase Conjugated) (1:5000, Cat:AP132P) was obtained from EMD Millipore Corporation. EEA1 (C45B10) Rabbit mAb (1:1000, Cat:3288T) and Cleaved Caspase-3 (Asp175) Antibody (1:1000, Cat:9661T) was obtained from Cell Signaling Technology. The dilution of all antibodies are mentioned in the manuscript.
Validation	All the antibodies used in this work were strictly followed the instructions provided by the manufacturers. All the antibodies have COA(Certificate of Analysis) and met the quality control standards defined by the manufacturers and have passed rigorous application-specific testing standards reported by the manufacturers.

Eukaryotic cell lines

Policy information about cell lines

Cell line source(s)	A549 cells were obtained from ATCC. H1299-ZsGreen1-p53R175H cells were generated and provided from Prof. Ge Shan at University of Science and Technology of China. And the H1299-ZsGreen1-p53R175H cells were make from H1299 cells (obtained from ATCC) that incorporated with p53R175H and ZsGreen1 genes. The details of H1299-ZsGreen1-p53R175H cells generation can be found in the report study (10.1073/pnas.1502159112).
Authentication	A549 and H1299 cells purchased from ATCC were authenticated by Short Tandem Repeat (STR) prior to purchase.
Mycoplasma contamination	Cell lines were not tested for mycoplasma contamination in the study.
Commonly misidentified lines (See ICLAC register)	No commonly misidentified cell lines were used in the study.
